# Sustainability of spatially distributed bacteria-phage systems

**DOI:** 10.1038/s41598-020-59635-7

**Published:** 2020-02-21

**Authors:** Rasmus Skytte Eriksen, Namiko Mitarai, Kim Sneppen

**Affiliations:** Niels Bohr Institute, University of Copenhagen, Copenhagen, Denmark

**Keywords:** Computational models, Ecological modelling, Microbial ecology, Bacteriophages

## Abstract

Virulent phages can expose their bacterial hosts to devastating epidemics, in principle leading to complete elimination of their hosts. Although experiments indeed confirm a large reduction of susceptible bacteria, there are no reports of complete extinctions. We here address this phenomenon from the perspective of spatial organization of bacteria and how this can influence the final survival of them. By modelling the transient dynamics of bacteria and phages when they are introduced into an environment with finite resources, we quantify how time delayed lysis, the spatial separation of initial bacterial positions, and the self-protection of bacteria growing in spherical colonies favour bacterial survival. Our results suggest that spatial structures on the millimetre and submillimetre scale play an important role in maintaining microbial diversity.

## Introduction

Naturally occurring bacteria often live in spatially structured habitats: in the soil^1,2^, in our guts^3,4^, and in food products^5^. The spatial heterogeneity is in part generated by the diversity of the microbial world^6^, in part by clusters of food sources, and in part caused by the fact that bacterial division leaves the offspring close to their “mother” and often lead to the formation of microcolonies^7–9^. The spatial heterogeneity may, in turn, amplify itself through the propagation of host-specific phages, if these percolate devastating infections through the parts of space with the most homogeneous distribution of their hosts^10^.

Traditionally, phage-bacteria ecosystems are modelled by generalized versions of the classical Lotka-Volterra equations^11–23^. In their simplest form, such mass action equations predict sustained oscillations which become damped when one takes into account resource limitations^20^. In contrast to the oscillating lynx-hare systems from macroscopic ecology^24^, the microbial ecology experiments appear much more damped^14,25^. In such microbial experiments, the addition of phages leads to an initial collapse of the susceptible bacterial population after which they ultimately grow to a high density which matches the steady-state prediction of the generalized Lotka-Volterra equations. However, with realistic parameters for phage infections^26^ and realistic bacterial starting populations, the Lotka-Volterra type equations predict that an invading phage typically causes a collapse of the bacterial population so large, that the bacterial population by most practical definitions goes extinct^23,27^. On long time scales, the bacteria will evolve additional defenses against the phage which will contribute to its long term survival, but first it must avoid eradication on the short time scale. Several solutions to the problem of bacterial coexistence have been suggested, most of which revolve around mechanisms that cause phage death. For example, it is possible that phages irreversibly bind to the bacterial debris left over from bacterial lysis and thus inactive them leading to more and more phage decay as the phage invasion takes hold^16,18^. Another solution is the inclusion variability in the adsorption rate of phages, e.g. by assuming two subspecies in a bacterial population, one with a high adsorption rate and one with a low adsorption rate^23,27^.

However, these suggestions have the same drawbacks as all mass action models, namely that they ignore the spatial constraints in the physical system. Consequently, the models fail to capture that propagating phages tend to systematically deplete nearby hosts and thus may be unable to reach more distant hosts before the phages decay. In our paper, we focus on spatial structures, both on the millimetre and submillimetre scale, and their ability to moderate the virulence of the phages.

A typical method for introducing space in bacteria-phage modelling is by the use of cellular automata models^10,28–31^. By utilizing a combination of lattices and diffusion of nutrient and phages, these models are used to study the interface between bacteria and phages in highly structured environments and have successfully addressed the importance of the spatial structures in various scenarios. However, many of them assume that one lattice site can contain at most one unit of bacteria, and only the bacteria adjacent to empty sites can replicate, limiting the growth to the surface of a cluster of bacteria^10,28–31^. This can be justified if one lattice site represents a cluster of cells that is enough to deplete the resource before it diffuses to the neighbouring sites. However, it has been shown that a microcolony can grow exponentially in volume for a substantial period of time^8,9^, demonstrating the importance of exponential growth even in a spatially structured environment.

With our model, we bridge the gap between the zero-dimensional mass action models and the spatial cellular automata models, and thereby incorporate both spatial structure and exponential growth of bacteria. This allows us to resolve length scales ranging from micrometres to more than centimetres while retaining (some of) the submillimetre behaviour of the cellular automata models. In particular, we are interested in systems where the bacteria form microcolonies, as is seen when bacteria grow in semisolid medium. We approximate the submillimetre structure of the colonies and retain exponential growth by making modifications to the traditional Lotka-Volterra models. It is worth noting that a similar approach of coupling mass-action growth and lattice model was taken in ref. ^32^ to simulate the phage attack on a biofilm with the spacial resolution of  ~ 4 μm. We here consider length scales which are orders of magnitude larger where one lattice site can contain several of microcolonies. This allows for faster and larger-scale simulations while including the effects of colony structure in the phage-bacteria interaction as described below.

**Figure 1:**
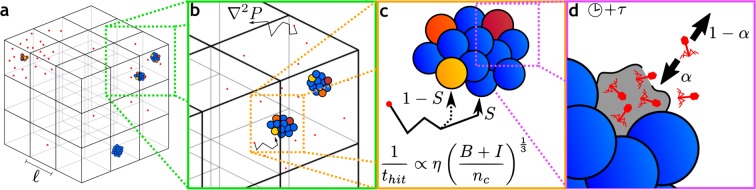
Schematic of new concepts. (**a**) Spatial variation is included by the introduction of a three-dimensional lattice where the dynamics of the bacteria-phage interaction plays out largely independently in the separate boxes. The initial bacteria and phages are randomly assigned to a box and accordingly, there will be some variation between the boxes. Over time these differences will amplify as the stochastic processes unfold. In some boxes, a lucky phage will adsorb early and quickly overwhelm the bacteria there, while in other boxes the phage might find the bacteria much later. (**b**) Within each box we imagine bacteria (coloured circles) growing as small colonies surrounded by the phages (red dots) and nutrient (not shown). If a box contains multiple colonies, they are assumed to be identical, i.e. we assume the colonies to be of equal size and with the same ratio of infected to uninfected cells. Diffusion couples the phages and nutrients in one box with neighbouring boxes and allows the system to equilibrate over time. (**c**) Adsorption to a single large target has different properties compared to adsorption to several small targets and consequently, the phage adsorption term is proportional to the bacterial density to the power of $\frac{1}{3}$. Furthermore, the submillimetre structure of the colony also modifies the probability for the phage to hit uninfected cells (parameterized by the function *S*). This structure is created by phages, which live outside the colony, adsorbing to the colony. In doing so, the phages will typically hit the outermost bacteria, while only rarely hitting the innermost cells. This discrepancy means the phages are more likely to be wasted to superinfecting the surface bacteria, and therefore not hitting the uninfected cells further in the colony. (**d**) Cell lysis occurs after a latency time of *τ* after which phages are released from the lysed cell. Since the lysed cell resides on the surface of the colony, the fate of these phages are different from the fate of the free phages surrounding the colony; of these progeny phages, we allow a fraction 1 − *α* to fully escape the colony and become free phages, while the remaining fraction *α* immediately find new targets to infect.

We partition space into a three-dimensional lattice, which allows for spatial variation in the densities of phage and bacteria (see Fig. 1(a)). Each box in the lattice is well-mixed, meaning that bacterial colonies within each box are identical, i.e. they have the same size and composition as each other, but they are typically different from colonies in other boxes. Due to their small size, phages and nutrients diffuse readily around in the system, while the much larger bacteria remain fixed in space. Consequently, the phages and the nutrients need to propagate before interacting with distant areas, and we include diffusion to couple the dynamics in one box with its neighbouring boxes (see Fig. 1(b)).

When bacteria form colonies the dynamics of phage interaction changes in several ways:

1. When phages adsorb to a single large target they do so with a rate proportional to the radius of the target as derived by Smoluchowski^33,34^, and thus proportional to the volume to exponent $\frac{1}{3}$. This reduces the adsorption rates to much smaller values than in unstructured situations (see Fig. 1(c)).
2. Infected bacteria typically lie on the surface of colonies rather than being uniformly distributed throughout the colony^9,28^. This means that the probability of a phage hitting an uninfected cell is not just proportional to the relative proportions of uninfected to total cells, but is reduced by the shielding function *S*, which we discuss in the methods section (see Fig. 1(c)).
3. When progeny phages are released from a colony, the phages will have many new targets readily available locally. We, therefore, include a readsorption effect where a fraction of the released phages immediately reinfect new bacteria while the remaining phages fully escape from the colony (see Fig. 1(d)).

Our model combines all of these aspects to describe phages attacking confined bacteria.

## Methods

A typical set of mass action equations for the bacterial density *B*, the density of free phages *P*, and the nutrient *n* take the form^13,14,23,35^:

1 $$\begin{array}{l}\frac{dn}{dt}=-\lambda B\frac{n}{n+K}\\ \frac{dB}{dt}=\lambda B\frac{n}{n+K}-\eta BP\\ \frac{dP}{dt}=(\beta -1)\eta BP-\delta P\end{array}$$

where the nutrient *n* is measured in units of bacteria it can be converted to (corresponding to a yield of 1), *λ* = 2 h^−1^ is the maximal bacterial growth rate, *η* = 10^−8^ mL/h is the adsorption rate for phages^26^, *β* = 100 is the phage burst size, and *δ* = 0.1 h^−1^ is the phage decay rate^36^. *K* = *n*(0 h)/5 is the Monod constant^37^ which determines at which concentration of nutrients the growth rate is halved. The initial nutrient level is set to *n*(0 h) = 10^9^/mL, meaning there is sufficient nutrient to produce 10^9^ bacteria within a millilitre of medium.

These equations are modified from standard Lotka-Volterra equations by the depletion of food and the associated parameterization of growth by the Monod equation. The above equations are the simplest way to model the development of a bacteria-phage system in a batch culture (e.g. as used for temperate phage dynamics in ref. ^35^).

Even before considering the effects of space on bacteria-phage interaction, the model noticeably ignores the latency time between phage infection and cell lysis. This is easy to include, either through a time delay equation^38^ or by introducing some intermediate infected states *I*_*i*_ between infection and lysis^39,40^. We choose the latter method and incorporate internal infected states *I*_*i*_.

2 $$\begin{array}{l}\frac{{\rm{d}}n}{{\rm{d}}t}=-\lambda B\frac{n}{n+K}\\ \frac{{\rm{d}}B}{{\rm{d}}t}=\lambda B\frac{n}{n+K}-\eta BP\\ \frac{{\rm{d}}{I}_{1}}{{\rm{d}}t}=\eta BP-\frac{10}{\tau }{I}_{1}\frac{n}{n+K}\\ \frac{{\rm{d}}{I}_{i}}{{\rm{d}}t}=\frac{10}{\tau }\frac{n}{n+K}\left({I}_{i-1}-{I}_{i}\right),\quad i=2,3,\ldots ,10\\ \frac{{\rm{d}}P}{{\rm{d}}t}=\beta \frac{10}{\tau }{I}_{10}\frac{n}{n+K}-\delta P-\eta (B+I)P\end{array}$$

The addition of latency time in Eq. 2 is obtained with the use of 10 intermediate infected states $I={\sum }_{i=1}^{10}{I}_{i}$ between infection and final release of phages^40^. The use of intermediate stages leads to the latency times being drawn from a gamma distribution with an average latency time *τ* = 0.5 h^26^ and a variation of 1/$\sqrt{10} \sim 30 \%$ between separate lysis events (see supplement for details). The virulence of phages is dependent on the growth conditions of the bacteria: in nutrient-poor media, the burst size and latency period of the phages are often smaller and longer respectively than in rich media^41–43^. It is not clear if the measured decrease in burst size is due to fewer infected cells reaching the lysis stage or if fewer phages are produced during the replication stage. In addition, it is well known that most phages cannot grow on stationary phase bacteria, with a few notable exceptions such as T7^44^. In our simulations, the transition from exponential phase to stationary phase is controlled by the amount of nutrient available. Therefore, we scale the latency between infection and lysis by the same Monod-like factor that controls bacterial growth. This means that as the nutrient is depleted, the latency time increases with bacterial generation time and thereby the phage invasion stops as the stationary phase is reached since no progeny phages can be released.

Notice that the addition of infected states reduces the virulence of phages not only by the introduced latency time, but by their mere presence. The phages do not discriminate between infected and uninfected bacteria so the phages also “waste” their genetic material by superinfecting the already infected bacteria. Note that the bacterial density, *B*, now refers to the uninfected bacteria, not the total bacterial density.

The above model corresponds to liquid conditions where the medium is continuously disturbed, e.g. by shaking. In this situation, the bacteria and phage are well-mixed and freely interact. If however the medium is left standing, then inhomogeneities may amplify and we use the spatiotemporal equation:

3 $$\begin{array}{l}\frac{\partial n}{\partial t}=-\lambda B\frac{n}{n+K}+{D}_{n}{\nabla }^{2}n\\ \frac{\partial B}{\partial t}=\lambda B\frac{n}{n+K}-\eta BP+{D}_{B}{\nabla }^{2}B\\ \frac{\partial {I}_{1}}{\partial t}=\eta BP-\frac{10}{\tau }{I}_{1}\frac{n}{n+K}+{D}_{B}{\nabla }^{2}{I}_{1}\\ \frac{\partial {I}_{i}}{\partial t}=\frac{10}{\tau }\frac{n}{n+K}\left({I}_{i-1}-{I}_{i}\right)+{D}_{B}{\nabla }^{2}{I}_{i},\quad i=2,3,\ldots ,10\\ \frac{\partial P}{\partial t}=\beta \frac{10}{\tau }{I}_{10}\frac{n}{n+K}-\delta P-\eta (B+I)P+{D}_{P}{\nabla }^{2}P\end{array}$$

The populations (*B*, *P*, *I*_1_, …, *I*_10_) and the nutrient *n* are now functions of space (*x*, *y*, *z*) as well as time, but we keep the parameters constant throughout. For readability, we don’t explicitly include the spatial dependency in the notation. In the model, *D*_*n*_, *D*_*B*_, and *D*_*P*_ are the diffusion constants for the nutrient, the bacteria, and the phages respectively. Here *D*_*n*_ ~ 10^6^ μm^2^/h^45^ is much larger than *D*_*P*_ ~ 10^4^ μm^2^/h^46,47^ and *D*_*B*_ ~ 5 ⋅ 10^2^ μm^2^/h^48^ (assuming no chemotaxis).

With these equations, we now model how delayed lysis and spatial heterogeneity in the population densities moderate the virulence of the phages. This limit corresponds to the situations where the bacteria are not immobilized and thus do not form colonies, e.g. as in standing liquid medium. The spatial heterogeneity then comes from the difference in diffusion rates where bacteria move substantially slower than the phages.

If the medium is more solid, the growth of the bacteria becomes even more spatially structured and submillimetre scale spatial structures related to bacterial colonies emerge. In these structured environments the bacteria are fully immobilized and do not diffuse (*D*_*B*_ = 0). Taken together, we present our full model for phage-bacteria interaction in a spatial context.

4 $$\begin{array}{l}\frac{\partial n}{\partial t}=-\lambda B\frac{n}{n+K}+{D}_{n}{\nabla }^{2}n\\ \frac{\partial B}{\partial t}=\lambda B\frac{n}{n+K}-S(B,I,{n}_{c},\zeta )\left[\eta {\left(\frac{B+I}{{n}_{c}}\right)}^{\frac{1}{3}}{n}_{c}P+\alpha \beta \frac{10}{\tau }{I}_{10}\frac{n}{n+K}\right]\\ \frac{\partial {I}_{1}}{\partial t}=S(B,I,{n}_{c},\zeta )\left[\eta {\left(\frac{B+I}{{n}_{c}}\right)}^{\frac{1}{3}}{n}_{c}P+\alpha \beta \frac{10}{\tau }{I}_{10}\frac{n}{n+K}\right]-\frac{10}{\tau }{I}_{1}\frac{n}{n+K}\\ \frac{\partial {I}_{i}}{\partial t}=\frac{10}{\tau }\frac{n}{n+K}\left({I}_{i-1}-{I}_{i}\right),\quad i=2,3,\ldots ,10\\ \frac{\partial P}{\partial t}=(1-\alpha )\beta \frac{10}{\tau }{I}_{10}\frac{n}{n+K}-\delta P-\eta {\left(\frac{B+I}{{n}_{c}}\right)}^{\frac{1}{3}}{n}_{c}P+{D}_{P}{\nabla }^{2}P\end{array}$$

The new concepts included in this model are shown schematically in Fig. 1 as a guide to the reader.

We investigate scenarios where the bacteria are constricted in the medium and remain effectively immotile. This situation is for instance encountered when performing experiments in soft agar^9^, but there are several other systems where the bacteria are constrained (such as in soil, biofilm, or solid foods). This constriction in space means that each initial bacteria grow to a colony of size (*B* + *I*)/*n*_*c*_, where *n*_*c*_(*x*, *y*, *z*) = *B*(*x*, *y*, *z*, 0 h) is the density of the initial bacteria and thus the density of colonies (see Fig. 1(b)), and *I* is the density of infected cells.

When bacteria grow in colonies, as opposed to growing separately, the dynamics of phage adsorption changes. Therefore, when a phage is adsorbed to one of the *n*_*c*_ colonies, it does so with a rate that is proportional to the colony radius^33,34^ (hence the $\frac{1}{3}$ exponent, see supplement for more details).

When some bacteria in the colony are already infected, then only a fraction *S* of the infections leads to new infected states, since the accumulated infected cells exist on the surface of the colony and partially shields the uninfected cell from the phage attack^9^. The remaining fraction 1 − *S* adsorb to these infected surface cells and consequently “waste” their genetic material to superinfecting bacteria instead of adsorbing to uninfected bacteria. This protection is defined by the shielding function *S*, which we will explain further below.

When lysis occurs, the released phage has a high probability of reinfecting the colony, which is represented by the parameter *α* = 0.5. A fraction (1 − *α*) of the phage progeny escape from the colony and becomes free phages *P*, while a fraction *α* readsorbs to bacteria in the colony. The functional form of the readsorption, as well as the value of *α*, is somewhat arbitrary, since we do not have a good model for what it means to “escape” a colony, nor do we have a model for how many phages should readsorb within a reasonable timescale. Instead, we use *α* as a simple way to penalize colony growth. In the supplement, we investigate several values of *α* and find that the survival of the bacteria is largely unchanging for *α* values between 0.5 and 0.95.

Returning to the choice of shielding function *S*, we investigated various shielding functions (see supplement for details), and found an approximate shielding function of the form:

5 $$\widetilde{S}(B,I,{n}_{c},\zeta )=\exp \left(-\frac{1}{\zeta }\left({\left(\frac{B+I}{{n}_{c}}\right)}^{\frac{1}{3}}-{\left(\frac{B}{{n}_{c}}\right)}^{\frac{1}{3}}\right)\right).$$

The derivation of this shielding function is inspired by particles moving through an absorbing barrier. Here the phages act as the particles and the barrier is the layer of infected cells, which has a thickness of ${r}_{0}\left({\left(\frac{B+I}{{n}_{c}}\right)}^{\frac{1}{3}}-{\left(\frac{B}{{n}_{c}}\right)}^{\frac{1}{3}}\right)$, where *r*_0_ is the radius of a single bacterium. The parameter *ζ* is the typical distance that a phage can penetrate before being absorbed (in units of *r*_0_). If the barrier is thinner than *ζ*, then most phage will pass through the barrier and vice versa. A nice feature of this shielding function is that there always is a non-zero chance of a phage slipping through the barrier. In the supplement, we estimate *ζ* as a function of the phage adsorption coefficient via detailed simulations of colonies consisting of individual spherical bacteria and find that for the diffusion-limited case *ζ* is roughly 1.

Note that to match in the shielding in the small colony limit where the probability of hitting an uninfected bacterium is just the fraction of uninfected bacteria in the colony (*S*(*B*, *I*, *n*_*c*_, *ζ*)$=\frac{B}{B+I}$), we must force an upper limit on the shielding:

6 $$S(B,I,{n}_{c},\zeta )=\min \ \left[\frac{B}{B+I},\ \widetilde{S}(B,I,{n}_{c},\zeta )\right]$$

In our implementation of the model, we use the above partial differential equations (PDEs) as a guide to design a discrete stochastic model on a lattice. The benefit of the stochastic model is that this more closely mimics the discrete nature of the bacteria and phages at the small scale. The solution to the PDEs will unfairly favour phage spreading since fractional infections of single bacteria will allow the phage population to increase earlier than would otherwise be the case. When the local number of bacteria or phages is small, our stochastic simulation include the associated fluctuations of populations between the boxes. When population numbers are persistently large, each process in the above equations occurs many times in each bacterial generation, and self-averaging over many events approximately eliminate any sign of stochastic variation.

We thus go from densities to integer populations and thereby track individual particles in the simulation. The only exception to this is the nutrients which we treat as a continuous field. Note that the use of integer populations allows for clearly defined extinction scenarios since the populations can reach zero members. In contrast, if using continuous densities, we would need to apply some threshold value for the populations to have well-defined extinction events.

We include the spatial variation by simulating a one cubic centimetre system where space is subdivided into a 3-dimensional lattice consisting of boxes of length *ℓ* = 0.2 mm. This approach allows for different numbers of bacteria and phages in each of the 1.25 ⋅ 10^5^ boxes in the lattice. We initialize the simulation by placing the individual bacteria and phages uniformly in the simulation space. Note that in order to represent the behaviour of a larger system we use periodic boundary conditions.

Inside each box in the lattice, we simulate our model stochastically by treating each term in Eqs. 1–4 as rates corresponding to events. At each time step, we compute the probability for every particle to undergo each event and draw the number of occurrences from a Poisson distribution (see supplement for details). Every box is simulated independently, modulated by diffusion of food and phages between the boxes. We treat each phage as performing a random walk on the lattice and during each time step we migrate appropriate fractions of the phage population to the neighbouring lattice boxes. Nutrient diffusion is computed by using a discrete Laplace operator (using the “forward time central space” scheme).

Since our lattice is resolved at a resolution of *ℓ* = 200 μm, we can approximate the bacteria as being effectively immotile (*D*_*B*_ = 0) since they diffuse on average  ~ 30 μm per hour and thus would be unlikely to travel much further than to the boxes immediately adjacent in the time frame we simulate. Note that if we were to resolve lattice at a much smaller resolution we would need to account for cell shoving dynamics which would redistribute the local bacteria into neighbouring boxes when the density approaches the capacity of the grid point.

The problem contains two different timescales, that of bacteria and phages versus that of nutrient diffusion, which is the fastest timescale. In order to solve nutrient diffusion accurately we require that ${D}_{n}\frac{\Delta T}{{\ell }^{2}}\le \frac{1}{6}$ (see supplement for derivation), which we obtain for time steps of size *ΔT* = 2 ⋅ 10^−3^ h ~ 7 s.

**Figure 2:**
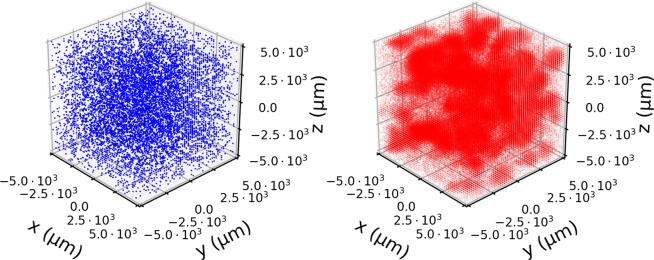
Snapshot of a simulation. The spatial distribution of bacteria (blue) and phages (red) at time *T* = 7 h starting from an initial *B* = 10^4^ bacteria per mL and *P* = 10^5^ phages per mL. To show the variation in densities, we scale the opacity of each point to be proportional to ${\rm{\log }}10$ of the population in that point. Despite uniform initial distributions, the stochastic dynamics cause large variation which is most clearly seen in the patchy distribution of phages.

In Fig. 2, we show an example of a single simulation. Here the spatial heterogeneity is evident both in the separation of bacteria colonies in the lattice (blue spheres) but also in the distribution of phages (red points). This is most clearly seen by the small “clouds” of phages diffusing away from areas where the invasion has taken hold.

Note that the model we present in Eq. 4 has Eq. 3 as a limiting case: If colonies do not form, then the number of colonies is equal to the number of bacteria $\left({n}_{c}=B+I\right)$ and the probability of hitting uninfected bacteria is equal to the fraction of uninfected to total bacteria $\left(S=\frac{B}{B+I}\right)$. In addition, the phages do not benefit from having adsorbed to the colony so *α* = 0. Furthermore, the model in Eq. 2 is a limiting case of Eq. 3. The only difference between these models is the inclusion of spatial variation.

The implementation of this model and the data generated by it can be found at the GitHub repository located at https://github.com/RasmusSkytte/SpatialBacteriaPhageModel/tree/v1.03

## Results

Our model is simulated with the four levels of temporal and spatial detail. Each level shows the effects of the changes from the initial mass action equations we presented in Eq. 1, to the full spatiotemporal model in Eq. 4 that also include protection due to colony formation.

**Figure 3:**
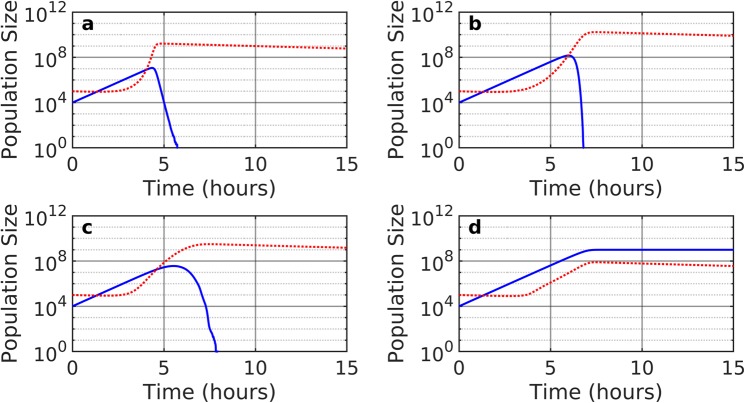
Dynamics of bacteria and phages. Here we show the time evolution of interacting phage and bacteria. The solid blue lines indicate the bacterial population, while the dotted red lines indicate the phage population. We show the time evolution for four levels of detail: (**a**) Well-mixed, Eq. 1. (**b**) Well-mixed with time delay, Eq. 2. (**c**) Spatial model with time delay, Eq. 3. (**d**) Spatial model with time delay and colony-level protection, Eq. 4. In all cases we start with the initial condition *B* = 10^4^ bacteria per mL and *P* = 10^5^ phages per mL, and assume a max production of *n*(0h) = 10^9^ bacteria per mL.

Examples of simulations are illustrated in Fig. 3, where one can follow the trajectories produced by the models: In (a) we simulate the simple well-mixed model in Eq. 1, and observe how the phage quickly overwhelms the bacteria leading to the extinction of the bacteria. In (b) we simulate Eq. 2, the well-mixed model that includes time-delay between phage infection and cell lysis. The latency of the lysis cycle delays the growth of phages, thereby leaving more time for bacteria to grow. As a result, the bacteria reach a higher density and subsequently is exposed to a more dramatic collapse when phage population catches up compared to panel (a). Thus the inclusion of the appropriate time delays predicts a more unstable behaviour and is accordingly less in agreement with typical batch growth experiments than the simpler equation used in panel (a). Panel (c,d) explores the two levels of spatial detail, both with the appropriate time delay due to latency. In panel (c) we simulate Eq. 3 which introduces spatial variation. The added heterogeneity of space further delays the extinction of the bacteria as different regions of space experience different phage densities. Panel (d) uses the full model in Eq. 4 that includes colony-level protection. One sees a dramatic improvement in stability, with bacteria surviving to reach a steady-state where normal phages cannot propagate. Notice that we do not model large plaque former like phages T7 here, as we assume that phage latency diverges with same Monod factor as the bacterial generation time (Eq. 4). In fact, when including protection due to colony formation, the bacteria can only be eliminated when the initial phage load is high enough to eliminate bacteria before they form sufficiently large colonies.

**Figure 4:**
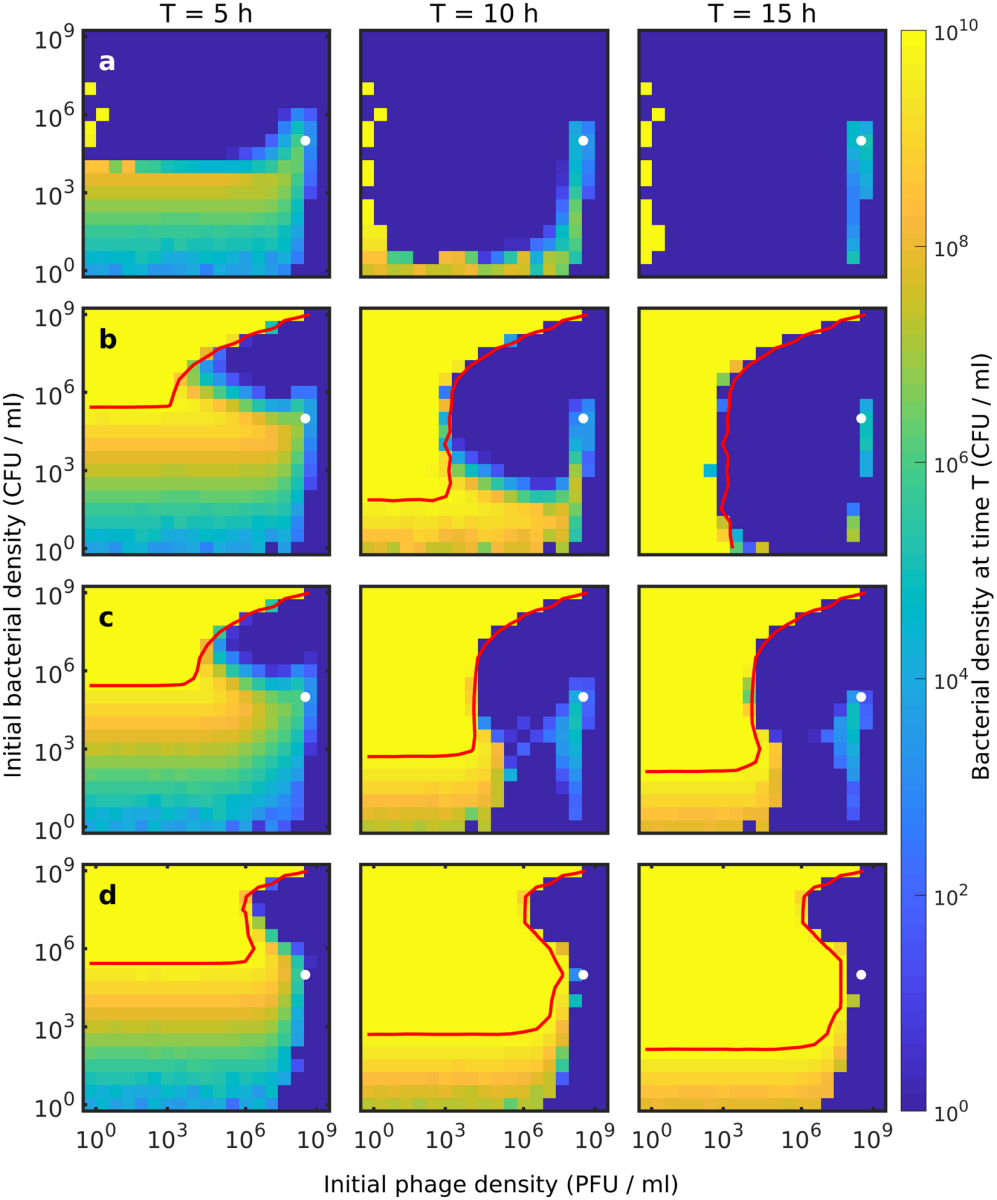
Phase space plots of the surviving bacteria. We investigate the outcome of the phage-bacteria interaction as a function of initial phage density and initial bacterial density. We show the bacterial density at the given time (averaged over the whole system) on a logarithmic colour scale ranging from bacterial extinction (blue) to the bacteria reaching the carrying capacity (yellow). Each column shows the dynamics at *T* = 5 h, *T* = 10 h, and *T* = 15 h respectively, while the rows indicate different models: (**a**) Well-mixed, Eq. 1. (**b**) Well-mixed with time delay, Eq. 2. (**c**) Spatial model with time delay, Eq. 3. (**d**) Spatial model with time delay and colony-level protection, Eq. 4. The red lines indicate the nutrient contour $\frac{1}{V}{\int }_{V}n\ dV=K$, where the growth rate is halved. For initial densities above this line, the nutrient is mostly depleted and the system reaches a steady-state. The white dots indicate where *P* = 2 ⋅ 10^8^/mL and *B* = 10^5^/mL. In the well-mixed model at these densities, the phages kill bacteria at the same rate as the bacteria divides, and the system is semi-stable around this point. In all four models, we observe similar patterns: When the phage load is low, the bacteria will survive and often consume all of the nutrients in most of the models (except for panel (a)). When the phage load is moderate to high, the time it takes for the phages to eliminate the bacteria varies. The bacteria are quickly overwhelmed when the initial densities are high, but as the initial densities are lowered, the time to extinction increases. In the phase space, this leads an emergent structure, where a “wave” of extinction moves clockwise around the white dot starting from the upper right corner. How deep into the phase space the extinction wave reaches before the nutrient is consumed decreases substantially as we include more and more protection mechanisms. At high phage loads  ~ 10^9^ PFU/mL or higher, the phage always overwhelm the bacteria in less than 5 h.

 Figure 4 explores bacterial survival as a function of the initial density of phages and bacteria in the four levels of modelling detail. In each case, the simulation is initiated with bacterial and phage populations between 1 and 10^9^ per mL. The spatial simulations consider 1 cm^3^ = 1 mL coarse-grained into boxes of dimension 0.2 mL, and a bacterial population of 10^5^ per mL accordingly correspond to an average population of about one bacterium per box. In each panel, one can see a dark blue region that represents conditions where bacterial populations are eliminated. For each model variant this “dead” region expands as simulation time is increased (going from left to right shows to the bacterial population at 5 h, 10 h, and finally 15 h). After 15 h the nutrient is depleted in most cases. This is highlighted by the red line which marks conditions where the average nutrient density is equal to the Monod growth constant: $\frac{1}{V}{\int }_{V}n\ dV=K$. For initial densities above and to the left of this red line, the accumulated number of bacterial divisions has consumed the bulk of the nutrient and these systems will subsequently remain largely unchanged.

Figure 4(a) show that in the traditional mass action models, the fate of the bacterial populations is similar across a large range of initial conditions where the phage eradicate the bacteria. In some cases, we observe transient survival where bacterial growth balances death due to phages. In quantitative terms, the bacteria population is marginally sustained when the rate of phage adsorbing to bacteria is approximately equal to the rate of bacteria division *λB* ~ *ηBP* or put differently:

7 $$P\approx \frac{\lambda }{\eta }=2\cdot 1{0}^{8}/{\rm{mL}}$$

Further, the phage population cannot increase when the rate of phage produced by lysis is less than the decay rate of the phage.

At the point where phage adsorption is balanced by the bacterial growth rate (*P* ≈ 2 ⋅ 10^8^/mL), the phage production rate is approximately:

8 $$\dot{P}\approx (\beta -1)\eta BP-\delta P$$

Which is zero when

9 $$B=\frac{\delta }{(\beta -1)\eta }\approx 1{0}^{5}/{\rm{mL}}$$

When the initial bacterial population is larger than 10^5^ CFU/mL, the phage population can grow from the beginning, whereas lower bacterial density means that phages cannot grow before the bacterial population has increased. Around these critical densities, the phage does not grow sufficiently fast to repress the bacterial population within 15 h.

Figure 4(a) shows that the initial Lotka-Volterra dynamics only predict long term survival when the phage density is extremely low, i.e. 1–3 *PFU*/mL. Since we simulate 1 mL of volume, we only have 1–3 phages in the simulation and these phages have a higher probability of decaying than they do of adsorbing to bacteria. In this case, the bacteria can consume all of the nutrients and thus survive indefinitely. Note, however, that this is a finite-size effect that disappears as the simulation volume is increased sufficiently.

Figure 4(b) demonstrate that the latency time of phage infection enables the bacterial population to survive in a much larger region of initial conditions, compared to panel (a). This survival arises because the latency allows the bacterial population to deplete the nutrient before the phage population grows large enough to exterminate the bacteria. This observation gives an addendum to our observation based on Fig. 3(b): while latency leads to larger crashes of the bacterial population in some cases, it leads to long term survival in other cases.

In Fig. 4(c,d) the initial conditions for long term survival for bacteria is greatly extended, demonstrating the moderating effects of space. The combination of spatial heterogeneity (c), and the buildup of sizable colonies (d) tend to provide for robust bacterial survival also in the region around 10^4^–10^8^ initial phage per mL.

**Figure 5:**
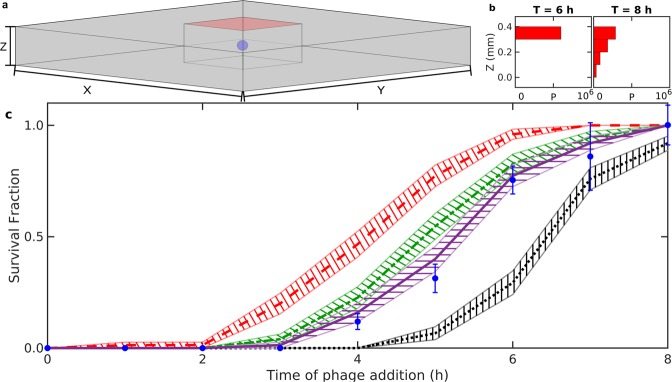
Reproducing experiment. We use our program to replicate the conditions of a previous experiment. Here, bacterial colonies grow inside a flat layer of soft agar for a set amount of time before phages were sprayed on the top of the soft agar. After an additional 16 hours, the fraction of surviving colonies was determined by counting visible colonies. (**a**) Illustration of a simulation. We show the spatial distribution of phages (red) and bacteria (blue) inside the soft agar (grey) at the time of phage addition. The colony is formed from a single starting bacterium and *P* = 6 ⋅ 10^5^ phages are distributed at *Z* = 400 μm at time *T* = 6.0 h to mimic phages being sprayed on the soft agar. Due to computational complexity, the phages are only resolved in a smaller region centred on the bacterial colony. (**b**) The vertical distribution of phages at time *T* = 6 h and *T* = 8 h showing how phages diffuse into the soft agar over time. (**c**) The fraction of colonies which grows to visible size in experiments 16 hours after phage exposure (blue error-bars)^9^ and the fraction of simulated colonies which produce more than 5 ⋅ 10^5^ cells after 16 hours for *ζ* = 2.5 (red dashed line), *ζ* = 5 (green dash-dotted line), and *ζ* = 10 (purple line). As a control, we include the prediction when the colony-level protections are disabled (black dotted line). The hatched area around each line indicates the standard error (*n* = 75). The simulation here uses P1 parameters^9,26^: *β* = 400, *δ* = 0.003 h^−1^, *η* = 1.32 ⋅ 10^4^ μm^3^/h, *τ* = 1 h, and parameters to match the experimental conditions^9^: Simulation volume of 400 μm × 10^4^ μm × 10^4^ μm, *D*_*P*_ = 3000 μm^2^/h (~1/10 of phage-*λ* value) and *λ* = 60∕31 h^−1^.

To test the model, we modify the setup to mimic the conditions used in the experiment performed in a recently published paper^9^. In their work, *Escherichia coli* was suspended in soft agar and plated onto Petri dishes. After a time where each bacteria had grown to some small colony, the plates were sprayed with the virulent phage *P*1_*vir*_. The number of visible bacterial colonies were counted 16 hours later. In the experiment, the formed bacterial colonies were about 1cm apart, embedded in a layer of soft agar of height  ≈ 400 μm. These conditions are replicated by simulating a single colony inside a box of dimensions 10^4^ μm × 10^4^ μm × 400 μm. The phages are spawned in the XY-plane at *Z* = 400 μm, and we change the boundary condition in the Z-axis from periodic to reflective (see supplement for full details). A visualization of the scenario is shown in Fig. 5(a). After the phage is released at *Z* = 400 μm, they slowly diffuse into the agar, creating a gradient of phage pressure with many phages at the top of the agar, and fewer towards the bottom. In Fig. 5(b), we show how the density of phage varies along the Z-axis at the time of phage addition (*T* = 6 h) and two hours later.

For each time *T*_*i*_ of phage spawning, we simulate 75 stochastic developments of a single bacterium forming a colony and compute the fraction that has grown to visible size at *T*_*i*_ + 16 hours. A colony is defined as visible if the number of produced cells is above 5 ⋅ 10^5^, corresponding to a colony of radius  ≈ 50 μm. Notice that we count cells produced and not the final alive cells, because dead cells also contribute to making the colony visible^9^. Operationally the number of dead cells is counted by measuring the consumed nutrient ∫_*V*_*n*(0h)d*V* − ∫_*V*_*n*(16h + *T*_*i*_)d*V* that was converted to biomass.

Figure 5(c) compare model prediction against the experimental observations^9^. When comparing the fraction of visible colonies in Fig. 5(c), the simulation with *ζ* = 10 reproduce the experimental result reasonably well, meaning that phages typically penetrate through 10 layers of bacteria before final adsorption.

In their paper^9^, the authors also derive a model of the critical radius *R*_*c*_ for a bacterial colony above which it can survive a phage attack. The model assumes the colony is a dense sphere and that the phages can penetrate a fixed distance *ΔR* into the colony. The derivation of our shielding function also assumes the colony to be a dense sphere of bacteria, which allows us to compare the penetration depth *ΔR* with our length scale *ζ*. We perform this comparison in the supplement and we obtain a value of *ζ* ≈ 8 bacterial radii.

## Discussion

The main prediction of this paper is that latency time and spatial structure greatly moderates the virulence of phages and thereby increases the robustness of phage-bacteria systems against dramatic collapses. This is in conceptual agreement with the constructed spatial study of ref. ^49^ and further suggests that even supposedly well-mixed environments^14^ may be structured on the microscopic scale. When latency time is included in the models, the phage attack is slowed and allows for bacteria survival in a relatively wide range of initial densities. Spatially varying densities improve this survivability and make bacteria survive up to 100-fold higher phage densities, while spatial structure on the level of microcolonies allows for survival with phage densities an additional 1000-fold higher. The geometry of the colonies reduces the phage adsorbance and shields susceptible bacteria in the centre of the colony against the invading phages. In the supplement, we investigate how space alone can help bacteria survive the phage attack, and find that space will increase survival substantially at low initial bacterial densities, but survival at high initial bacterial densities is governed by the latency time between phage infection and cell lysis.

Our investigation suggests that lessons learned in a well-mixed experimental system will translate poorly to scenarios where spatial heterogeneity is intrinsic. Even if the bacteria do not form microcolonies (Fig. 4(c)), the bacteria will easily survive 10-fold higher phage densities. We tested scenarios where phage and bacteria are initially uniformly distributed in space and note that if the initial distribution is more heterogeneous, the bacteria may survive even higher phage densities. One important aspect of the increased survivability of the bacteria when forming colonies is the fundamental change to the adsorption kinetics where clustered bacteria acts as fewer, but larger, targets.

Intuitively the fate of bacteria under a phage attack is reliant on their ability to postpone the phage attack long enough for the bacteria to consume all the available nutrient. For this reason, we see a big difference when we account for the latency time *τ* (see Fig. 4(b)), since here the phage invasion is slowed, allowing more time for the bacteria to consume the available nutrient. Spatial heterogeneity further slows the phage invasion as the diffusion of particles acts as an additional time delay mechanism. As the consumption of the available nutrient can be considered a refuge for the bacteria, this suggests that a viable strategy is to grow as fast as possible, since nutrient diffuses faster than phages throughout the system. This means that even if parts of the space is completely dominated by phages, nearby bacteria might consume the common nutrient and thereby prevent the phages from fully collapsing the heavily infected areas.

Figure 4 highlight two scenarios which lead to the elimination of bacteria. In the language of phage therapy, these situations correspond to “passive” and “active” therapy^50^. When the concentration of phages is above ~10^8^ PFU/mL the phage load is high enough to kill the bacteria within the first few bacterial generations, corresponding to the effects of “passive” therapy where the initial phage load eliminates the bacteria. Conversely, “active” therapy corresponds to the observed behaviour of the region of the phase space which is eradicated between *T* = 0 h and *T* = 15 h. Here the phages require multiple replication rounds before finally eliminating the bacteria.

When the concentration of colonies becomes small (below 10^3^ CFU/mL), the distance between colonies become large enough that limitations of nutrient diffusion stagnates total growth (Fig. 4(c,d)). Even after 15 hours, the nutrient is not fully consumed (as indicated by the red contour line). In the well-mixed cases (Fig. 4(a,b)) this stagnation is not seen since nutrient does not have to travel and is instantly available to the bacteria. Further, when the inoculation density is this low, the colonies would reach sizes above of 10^6^ cells and micro-gradients *within* the colony might slow growth even more than our model accounts for^8^.

Our study was simplified in multiple ways. In particular, we considered the survival of one bacterial strain exposed to one phage strain. This implicitly ignores nutrient depletion due to competing bacteria, a situation which typically will favour the long term survival of any particular strain of bacteria. Our model also ignored the fraction of bacteria that are resistant against a given phage^51^. If there is a fraction of about 10^−5^ to 10^−6^ bacterial mutants that lack the surface receptor used by the phage, a bacterial collapse will of course not be complete.

We have found no published experiments on phage-bacteria interactions that reported cases where the susceptible bacteria are completely eliminated. In this perspective, the presented modelling is a step towards a better explanation, although it still predicts conditions where bacteria should be completely eliminated. Thus further modifications to our modelling could be needed, for example by allowing the phage infections to vary hugely between the different bacteria. Perhaps the widely acclaimed variability in bacterial gene expression is a desired trait, that allows some bacteria to be resistant to phages for a substantial number of generations^52–54^.

Our equations (Eq. 4) are an approximated treatment of a system which can have quite variable strain-specific properties. For example, our diverging latency time with nutrient depletion means that the phages do not prey on stationary state bacteria, an assumption that is mostly correct but would fail for example for phage T7 preying on *Escherichia coli*^44^. Note that spatial structure is still relevant in such a case, as shown in protection by biofilm that hinders physical contact of phages to bacteria^30,32,55^. We use a relatively high decay rate for the phage, *δ* = 0.1 h^−1^ which is comparable to the larger values reported in the ocean^36^ but significantly higher than what is reported in more stable media^26^. However, the dynamics in our investigation typically play out on a relatively short time scale of ~10–15 h, meaning that phage decay is not likely to change the results substantially. The only exception to this claim, is for initial densities around the semi-stable point at $P\approx \frac{\lambda }{\eta }$ and $B\approx \frac{\delta }{(\beta -1)\eta }$. The depletion of the nutrient is an important factor in our model, however, we do not consider the possibility of nutrients being released upon lysis of bacteria. In this case, we expect that the increase in nutrients around the colonies under phage attack will prolong the duration of the attack and lead to higher phage densities but not substantially move the transition from survival to extinction. The equations also do not consider delayed lysis, where for example the simultaneous infections by several T4 phages cause longer latency times^56^. Furthermore, the equations do not consider temperate phages, which would only induce a collapse of order 0.1 to 0.01 (typically recorded lysogeny frequencies^40^). Finally, the treatment of bacterial colonies is simplified, as we have not yet obtained a complete understanding of their protective nature^9^.

A major factor in the strength of the protection mechanism is the parameter *ζ* which is a measure of the typical penetration depth of the phages. The ability of phages to penetrate bacterial colonies is likely to be dependent on several factors such as the geometry of the cells, the packing densities of the colonies, and whether or not the cells form a biofilm. Thereby this part of the phage protection should depend on the ability of the bacterial species to quorum sense and to collectively modify its local environment.

The model highlights the importance of colony formation and their ability to provide safe shelters. In fact, given that increasing colony sizes provide both local protection and make phage percolation between colonies less likely, one should consider colony formation as a strategic option for both bacteria in the wild, our use of bacteria in industry or in human gut reimplantation, and in situations where one wants to eliminate pathogenic bacteria by phage therapy.

## Supplementary information

Supplementary Information.
