# Supplementary material for: Sustainability of spatially distributed bacteria-phage systems
Source: Sci Rep. 2020 Feb 21;10:3154. doi: 10.1038/s41598-020-59635-7 (PMC7035299; doi:10.1038/s41598-020-59635-7)
Supplement: Supplementary file 1 — Supplementary Information. [file 41598_2020_59635_MOESM1_ESM.pdf]

Supporting Information. Sustainability of spatially distributed bacteria-phage systems

Rasmus Skytte Eriksen[1], **Namiko Mitarai**[1], **and Kim Sneppen**[1,*]

[1]Niels Bohr Institute, University of Copenhagen, Copenhagen, Denmark.
[*]sneppen@nbi.ku.dk

Contents

1 Adsorption to a single large target **2**

2 The spatial and temporal resolution **3**

3 The choice of shielding function **4**

4 The readsorption parameter **8**

5 The mechanisms of the colony-level protection **9**

6 The effect of spatial heterogeneity in the full model **12**

7 The distribution of the latency time **13**

8 The effect of latency in the full model **15**

9 Simulating experimental conditions **16**

9.1 Comparing ΔR with ζ . . . 16

9.2 Phage diffusion constant and the colony survival fraction . . . 16

10 Implementation details **18**

11 Von Neumann stability analysis of the 3D FTCS scheme **20**

References **20**

1 Adsorption to a single large target

A key component of our full spatial model is a modification to how adsorption works on confined bacteria. When bacteria are not confined, each cell acts as a small spherical sink for the phages. However, when bacteria are confined we can consider the colony as a whole to act as a spherical sink. This difference is important, as bacteria in the centre of the colony are not interacting with the phages. Mathematically, we can use a result derived by Smoluchowski[1], where he solved the rate k at which small particles, diffusing with diffusion constant D, adsorbs onto a spherical sink of radius b:

$$k = 4\pi Db$$

This derivation assumes that the target is sufficiently large to be assumed stationary relative the the smaller diffusing particle. For phages and bacteria, this assumption is a good one since the diffusion constant of the phages, $D_P \sim 10^4$ μm^2/h[2,3], is much larger than that of the bacteria, $D_B \sim 5 \cdot 10^2$ μm^2/h[4]. Note that this assumption would not hold if the bacteria are actively moving as by chemotaxis or other means.

If we consider the single bacterium to have radius r_0, then a colony containing $B+I$ members must have a volume of $V_c = \frac{4\pi}{3} r_c^3 = (B+I)\frac{4\pi}{3} r_0^3$. This means that the radii r_c and r_0 are related by:

$$\frac{r_c}{r_0} = (B+I)^{1/3}$$

The rate of a phage adsorbing to a *bacterium* is then equal to: $\eta = 4\pi D r_0$. While the rate of phage adsorbing to a *colony* is equal to:

$$\begin{aligned}\eta_c &= 4\pi D r_c \\ &= 4\pi D (B+I)^{1/3} r_0 \\ &= \eta (B+I)^{1/3}\end{aligned}$$

2 The spatial and temporal resolution

Due to the large diffusion constant of the nutrient in our simulations, we need to run the simulation at very small temporal resolution in order for the solution to be numerically stable (see Section 11 for details). This limits our spatial resolution and we, therefore, run the simulations in our paper using $\ell = 200$ µm, which corresponds to a lattice of $50 \times 50 \times 50$ boxes. Based on the derivation in Section 11, we use a time step of $\Delta T = 2 \cdot 10^{-3}$ h. In Fig. S1, we test how well the simulation results have converged using model 4 (equation (4) of the main text) as a reference. With model 4, we test different spatial resolutions and different time steps.

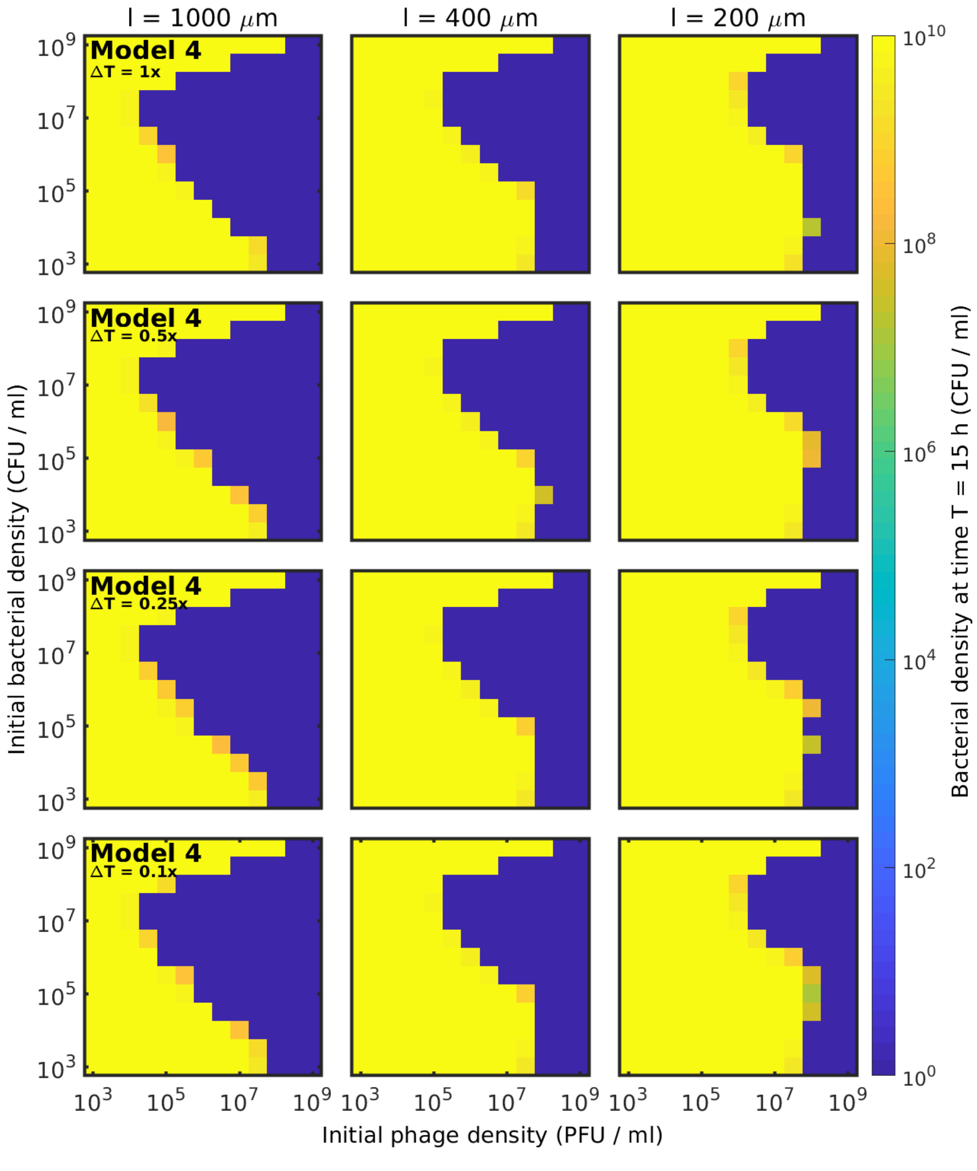

Figure S1. Testing spatial and temporal resolutions. We show how the resolution of the lattice changes the simulation outcomes of model 4 (equation (4) in the main text) using four different box sizes: $\ell = 1000$ µm, $\ell = 400$ µm, and $\ell = 200$ µm. These box sizes corresponds to lattices of dimensions $10 \times 10 \times 10$, $25 \times 25 \times 25$, and $10 \times 50 \times 50$ respectively. For each value of ℓ, we run the model with increasingly small time steps ranging from $\Delta T = 2 \cdot 10^{-3}$ h to one tenth this value. (Note the change in limits on the axes compared to the other figures of this type)

We see that the relative change going from $\ell = 400$ µm to $\ell = 200$ µm is smaller than when going from $\ell = 1000$ µm to $\ell = 400$ µm which suggest that the simulation is converging and that the results will not change drastically when going to a smaller resolution. In addition, we see that decreasing the time step only weakly influences the results.

3 The choice of shielding function

When bacteria grow as a colony they only expose the outermost layers to the outside environment. Invading phages are therefore more likely to interact with these outermost bacteria rather than the innermost bacteria which are shielded from the phages by the surface bacteria. The shielding function S represents the reduced probability of a phage hitting an uninfected cell when bacteria grows in colonies as compared to the well-mixed scenario. To build evidence for our choice of shielding function, we ran agent-based simulations of microcolonies using the framework described in ref.[5] which you can find at the following link: https://github.com/RasmusSkytte/GrowingMicrocolonyProtection/tree/v1.0.

This framework simulates individual colonies by treating each bacterium as a spherical particle. Using this framework, we have simulated phage adsorbing to microcolonies of a various sizes ($N = \{10, 32, 100, 316, 1000\}$) and with different fractions ($f = \{20\%, 40\%, 60\%, 80\%\}$) of infected cells situated at the surface of the colony. For each microcolony and each fraction of infected cells, we run 50.000 simulations where a single phage adsorbs to the colony. By counting the number of runs that result in an uninfected bacteria being hit, we get the probability that the phage is shielded by the layer of infected cells. We then compare four different shielding functions against this data.

The simplest shielding function we use is constructed by treating the infected cells as small disks residing on the surface of a spherical colony of radius r_c. Each infected cell covers a portion of the colony which we take to be an area of size πr_0^2, where r_0 is the radius of a single cell. If the colony is entirely covered by the infected cells all phages are shielded.

If we define A_I to be the area covered by infected cells, we can define the probability of a phage passing through the layer of infected cells as: $p = 1 - \frac{A_I}{4\pi r_c^2}$.

In a given grid point, we have n_c colonies, B uninfected bacteria and I infected bacteria. This means that each of the n_c colonies will have a volume: $V = \left(\frac{B+I}{n_c}\right)\frac{4\pi r_0^3}{3} = \frac{4\pi r_c^3}{3}$, while the area covered by the infected bacteria is $A_I = \frac{I}{n_c}\pi r_0^2$. By rearranging the equation for the volume, we get the following relation: $r_0^2\left(\frac{B+I}{n_c}\right)^{\frac{2}{3}} = r_c^2$.

We now plug these equations into the equation for p to get the more detailed expression:

$$p = 1 - \frac{A_I}{4\pi r_c^2} = 1 - \frac{\frac{I}{n_c}\pi r_0^2}{4\pi r_0^2\left(\frac{B+I}{n_c}\right)^{\frac{2}{3}}} = 1 - \frac{I}{4n_c}\left(\frac{n_c}{B+I}\right)^{\frac{2}{3}}$$

In the limit where the colonies are very small, the probability of hitting an uninfected cell should just be the ratio of uninfected to total cells, i.e. $p = \frac{B}{B+I} = 1 - \frac{I}{B+I}$. In order to match our shielding function with this limit, we drop the factor of 4 and we are left with the shielding function:

$$S_1(B, I, n_c) = 1 - \frac{I}{n_c}\cdot\left(\frac{n_c}{B+I}\right)^{\frac{2}{3}} \tag{S1}$$

For this shielding function however, we only match the small colony limit when the colony consists of a single bacterium.

Another way of matching this limit and retaining the factor of four in the expression above, is by using a piece-wise function. Here we keep the factor of 4 when $(B+I)/n_c > 64$, and use a linear term when $(B+I)/n_c \leq 64$. This gives our second shielding function:

$$S_2(B, I, n_c) = \begin{cases} 1 - \frac{I}{n_c}\cdot\left(\frac{n_c}{B+I}\right) & (B+I)/n_c \leq 64 \\ 1 - \frac{I}{4\cdot n_c}\cdot\left(\frac{n_c}{B+I}\right)^{2/3} & (B+I)/n_c > 64 \end{cases} \tag{S2}$$

Instead of considering the area covered by the infected cells, we next consider the volume of the infected cells occupy: $V_I = \frac{I}{n_c}\frac{4\pi r_0^3}{3}$. If we assume that a phage can penetrate a maximum distance kr_0 into the colony, we can define the probability of a phage passing through the infected bacteria as the ratio of the volume of the infected cells to the volume of a shell of thickness kr_0 located on the surface of the colony:

$$p = 1 - \frac{V_I}{\frac{4\pi}{3}(r_c^3 - (r_c - kr_0)^3)} = 1 - \frac{\frac{I}{n_c}r_0^3}{r_c^3 - (r_c - kr_0)^3}$$

From before, we know that: $r_0\left(\frac{B+I}{n_c}\right)^{\frac{1}{3}} = r_c$.

$$p = 1 - \frac{\frac{I}{n_c}}{\left(\frac{B+I}{n_c}\right) - \left(\left(\frac{B+I}{n_c}\right)^{\frac{1}{3}} - k\right)^3}$$

Again, we want the shielding function to match with the small colony limit, so we obtain the shielding function $S_3(B,I,n_c,k)$:

$$S_3(B,I,n_c,k) \quad = \min\left(\frac{B}{B+I}, 1 - \frac{\frac{I}{n_c}}{\frac{B+I}{n_c} - \left(\left(\frac{B+I}{n_c}\right)^{1/3} - k\right)^3}\right) \tag{S3}$$

The above shielding functions all consists of hard cutoffs, in the sense that once the infected bacteria cover sufficient area/volume the shielding is perfect and no uninfected bacteria can be hit. This is not a very realistic description, as there will always be a small chance of a phage slipping through the cracks and hitting the uninfected bacteria within. For our final shielding function, we switch to a softer shielding function where we treat the phages as particles moving through an absorbing barrier. This barrier is the layer of infected bacteria which has a thickness of $d = r_c - r_u$, where r_u is the radius of the uninfected core. The phage has a constant probability of being absorbed as it moves through the barrier leading to an exponentially decaying probability of the particle passing through the barrier, but there is always a non-zero probability of successfully hitting an uninfected bacterium.

If we assume the barrier starts at $x = 0$, and that the phages are absorbed on a characteristic length-scale of $\tilde{\zeta}$, then the density of phages passing inside the barrier obeys:

$$\frac{\mathrm{d}P}{\mathrm{d}x} = -\frac{P}{\tilde{\zeta}}$$

Integrating yields the density curve:

$$P = P_0 \exp\left(-\frac{x}{\tilde{\zeta}}\right)$$

The probability of the phage passing through the barrier is then computed by converting the density curve to a probability density and taking the integral of the probability density over the barrier:

$$\begin{aligned}
p &= 1 - \frac{1}{\tilde{\zeta}} \int_0^d P \,\mathrm{d}x \\
&= 1 + \left[\exp\left(-\frac{x}{\tilde{\zeta}}\right)\right]_0^d \\
&= \exp\left(-\frac{d}{\tilde{\zeta}}\right) \\
&= \exp\left(-\frac{r_c - r_u}{\tilde{\zeta}}\right) \\
&= \exp\left(-\frac{r_0}{\tilde{\zeta}}\left(\left(\frac{B+I}{n_c}\right)^{1/3} - \left(\frac{B}{n_c}\right)^{1/3}\right)\right)
\end{aligned}$$

If we define $\zeta = \frac{\tilde{\zeta}}{r_0}$, then we get our final shielding function:

$$S_4(B,I,n_c,\zeta) \quad = \min\left(\frac{B}{B+I}, \exp\left[-\frac{1}{\zeta}\left(\left(\frac{B+I}{n_c}\right)^{1/3} - \left(\frac{B}{n_c}\right)^{1/3}\right)\right]\right). \tag{S4}$$

Here we also have to take the minimum to match the small colony limit.

In Fig. S2 we show how each of the shielding functions match with the microcolony simulations when $\eta = 10^4$ µm^3/h.

Based on this analysis, we choose to use S_4 as the model of the shielding effect. Continuing with this model, we investigate in Fig. S3 how the parameter ζ changes as a function of the adsorption coefficient. We again use our agent-based microcolony simulations to generate the data and fit the model to this data using least-square fitting.

The value of ζ extracted from this analysis ($\zeta \approx 1$), is not consistent with the value we find in the simulation of the experiment ($\zeta \approx 10$). This discrepancy is likely due to how the cells pack in the agent-based simulations. In these simulations, we do not include any diffusion of the bacteria, meaning that they always pack tightly. In addition, the cells in the simulations are spherical instead of elongated, which might influence the packing further.

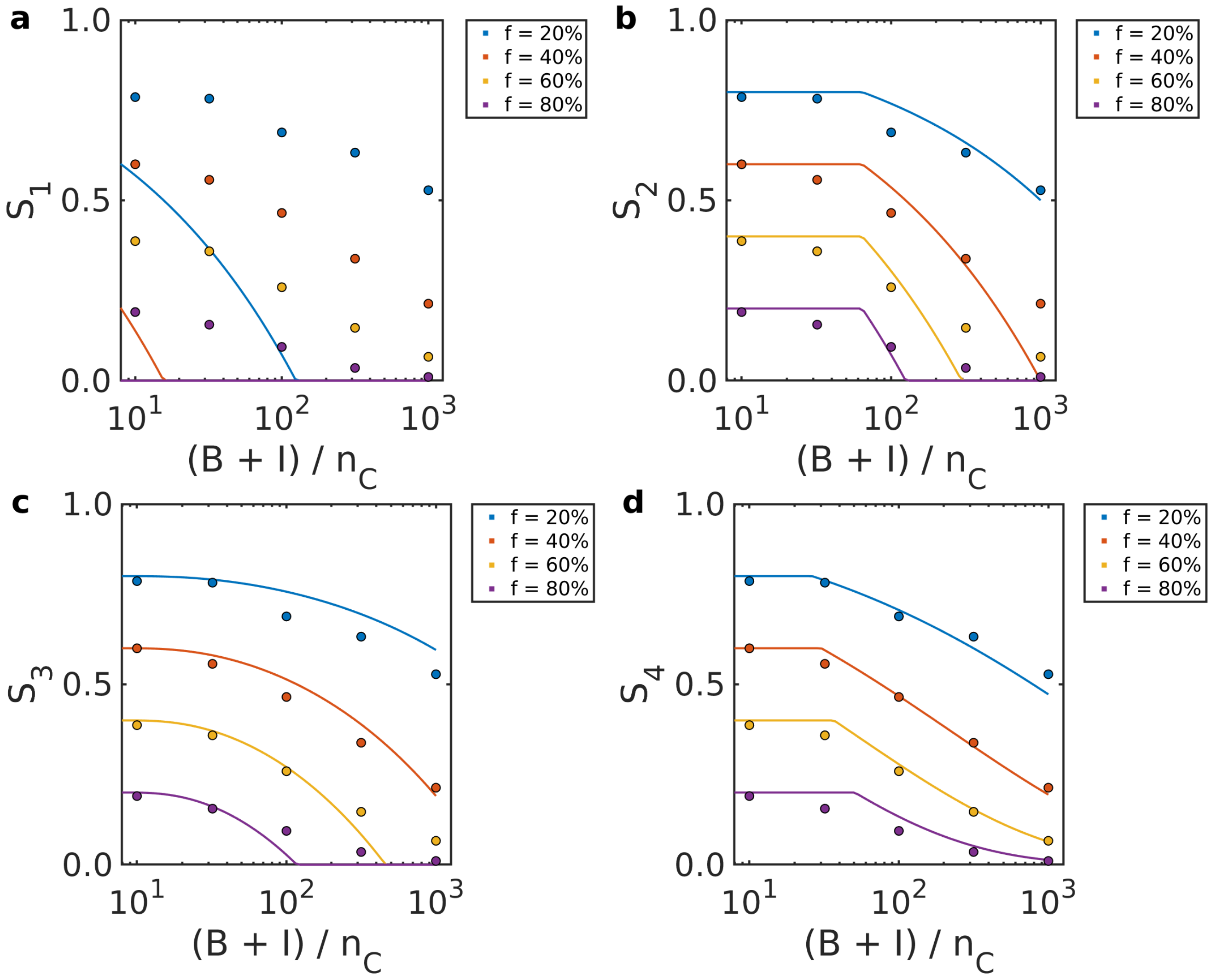

Figure S2. Testing shielding functions. We tested four different shielding functions against simulated data for $\eta = 10^4\ \mu\text{m}^3/\text{h}$ (circles). (a) S_1. (b) S_2. (c) S_3. (d) S_4. For each shielding function we visualize its shape using contours after using least-square fitting on the models with parameters (S_3 and S_4).

A value of ζ close to 1, corresponds with a penetration depth of one cell radius or equivalently, a penetration of $\Delta R \approx 0.6\ \mu$m. Which means it corresponds to the diffusion-limited case where the phage adsorbs to the first target they meet.

In Fig. S4, we show the difference in bacterial survival between two values of ζ in model 4 (equation (4) of the main text).

We see that when $\zeta = 10$, a large part of the phase space goes extinct when compared to the scenario where $\zeta = 1$. The reason is that the shielding is weaker and therefore bacteria do not establish colonies of sufficient size before the phage overwhelm them.

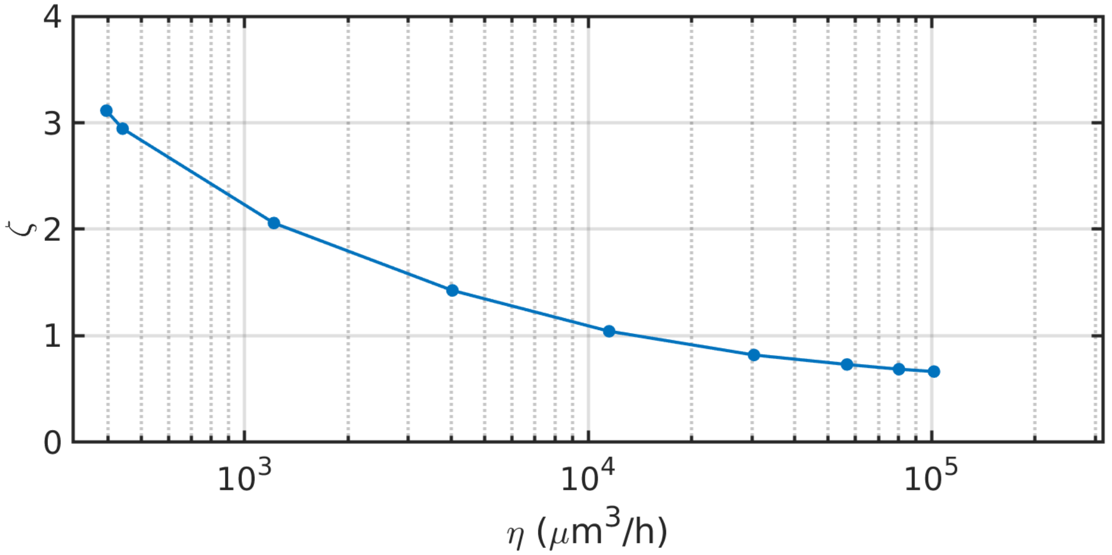

Figure S3. Coupling ζ to adsorption rate. We simulated phage adsorbing to microcolonies for different adsorption rates η, which changes how deep into the colonies the phage move on average before adsorbing. Using our shielding function S_4, we fit the parameter ζ to each of these data sets to determine their relation.

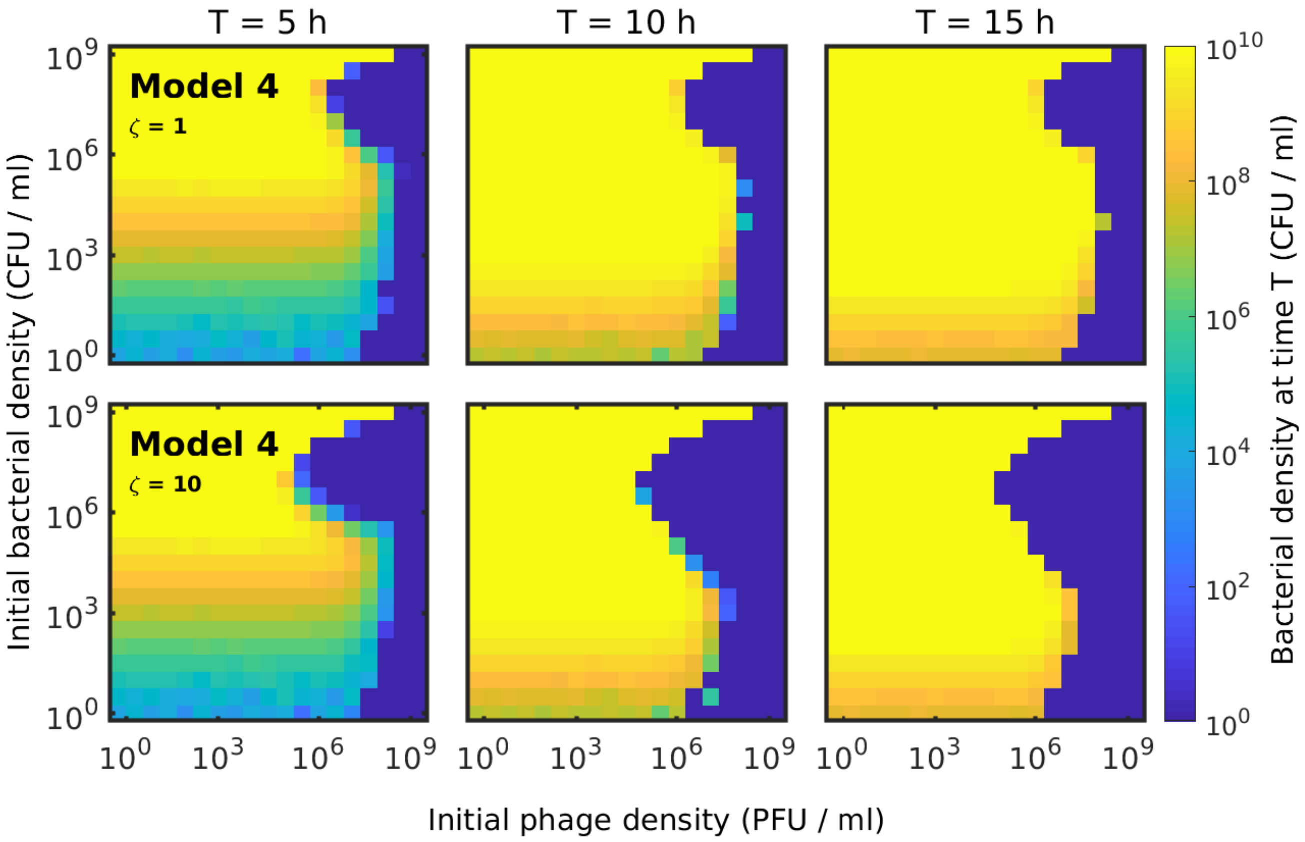

Figure S4. Testing phage penetration depths. We investigate how the penetration depth ζ influences the ability of colonies to survive the phage attack in model 4 (equation (4) in the main text). The value of ζ can be thought of as the number of layers of bacteria the phage typically moves through before adsorbing. $\zeta = 1$ corresponds was used in Fig. 4(d).

4 The readsorption parameter

In model 4 (equation (4) of the main text), the progeny phages have a probability α of immediately readsorbing to the colony and infect new cells. We do not have an estimate for what value this parameter should have, as it reflects a complicated stochastic process of how long it takes a diffusion particle to disassociate with an area of space.

We use the value $\alpha = 0.5$ as a rough estimate, and in Fig. S5 we compare with the limiting cases: $\alpha = 0$ and $\alpha \sim 1$.

When α is near zero, the bacteria are not penalized for growing as a colony and are therefore very likely to survive. This scenario is not realistic as any phage progeny released by lysis in a colony should lead to the new phages quickly finding the nearby bacteria.

As α approaches 1, we have the reverse scenario, where progeny phages always find the colony from which they originated. This means that the density of free phages never increases, and consequently, any colony which is not hit by the original phages are never going to be hit by any phages, which again increases the likelihood of colony survival.

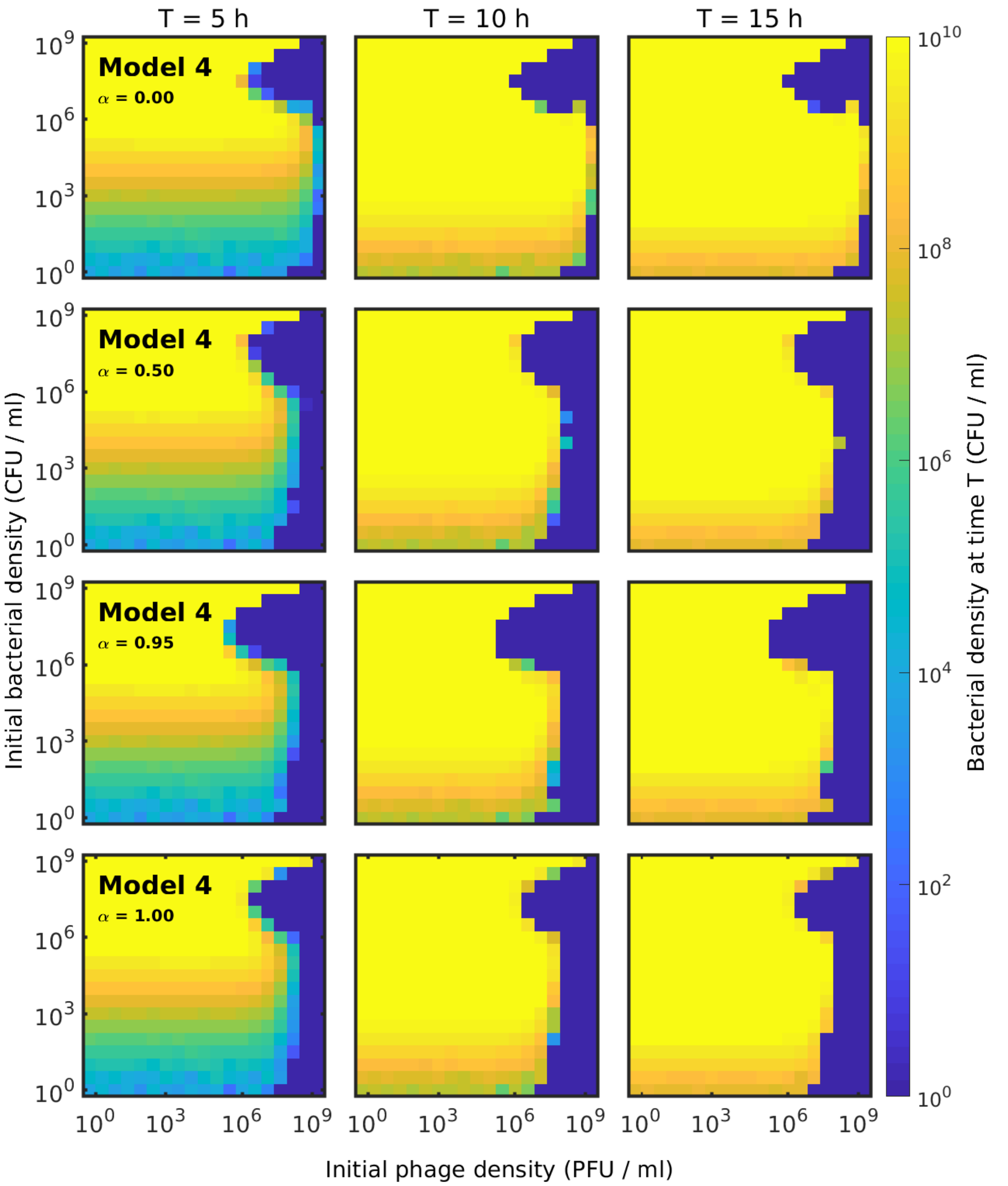

Figure S5. Testing phage readsorption. We run simulations of model 4 (equation (4) in the main text) where we change the fraction of phages which is immediately readsorped upon lysis. By testing values of α from $\alpha = 0$ to $\alpha = 1$, we change from a scenario where all phages become free phages, to a scenario where all phages are recaptured by the colony immediately. $\alpha = 0.5$ was used in Fig. 4(d).

5 The mechanisms of the colony-level protection

Going from the spatial Lotka-Volterra model (equation (3) in the main text) to the full model with colony-level protection in (equation (4) in the main text) added several mechanisms which influence the outcome. We here test how each of these mechanisms affects the colonies ability to survive the phage attack. The colony-level protection consists of three parts:

1. Clustering
2. Shielding
3. Readsorption

Clustering was introduced as a reduction in the effective adsorption rate. That is, we exchanged the well-mixed term ηB with the term $\eta \left(\frac{B+I}{n_c}\right)^{\frac{1}{3}} n_c$. Shielding was introduced via the function $S(B, I, n_c, \zeta)$, and readsorption is controlled by the parameter α. Each of these mechanisms can be controlled individually, and we can test their influence one at a time. In Fig. S6, we show what each mechanism does on its own when added to model 3 (equation (3) of the main text).

It is clear that, when taken alone, the clustering has the biggest effect on survival, but the shielding effect also provides significant protection. Interestingly, the readsorption effect does not on its own change the survival significantly in most cases, but it does remove the marginally stable region around $P = 2 \cdot 10^8\ PFU/\text{mL}$ and $B = 10^5\ CFU/\text{mL}$.

Next, in Fig. S7, we show how removing each of mechanism changes the survival of model 4 (equation (4) of the main text).

Here the antagonistic influence of the readsorption shows strongly. Neither the shielding or the clustering effects can on their own improve survival significantly when the readsorption is included. In both cases, we see that readsorption cancels out the effect of the protection mechanism (clustering / shielding). When readsorption is removed, however, the two protective mechanisms add up to yield a huge area of survival.

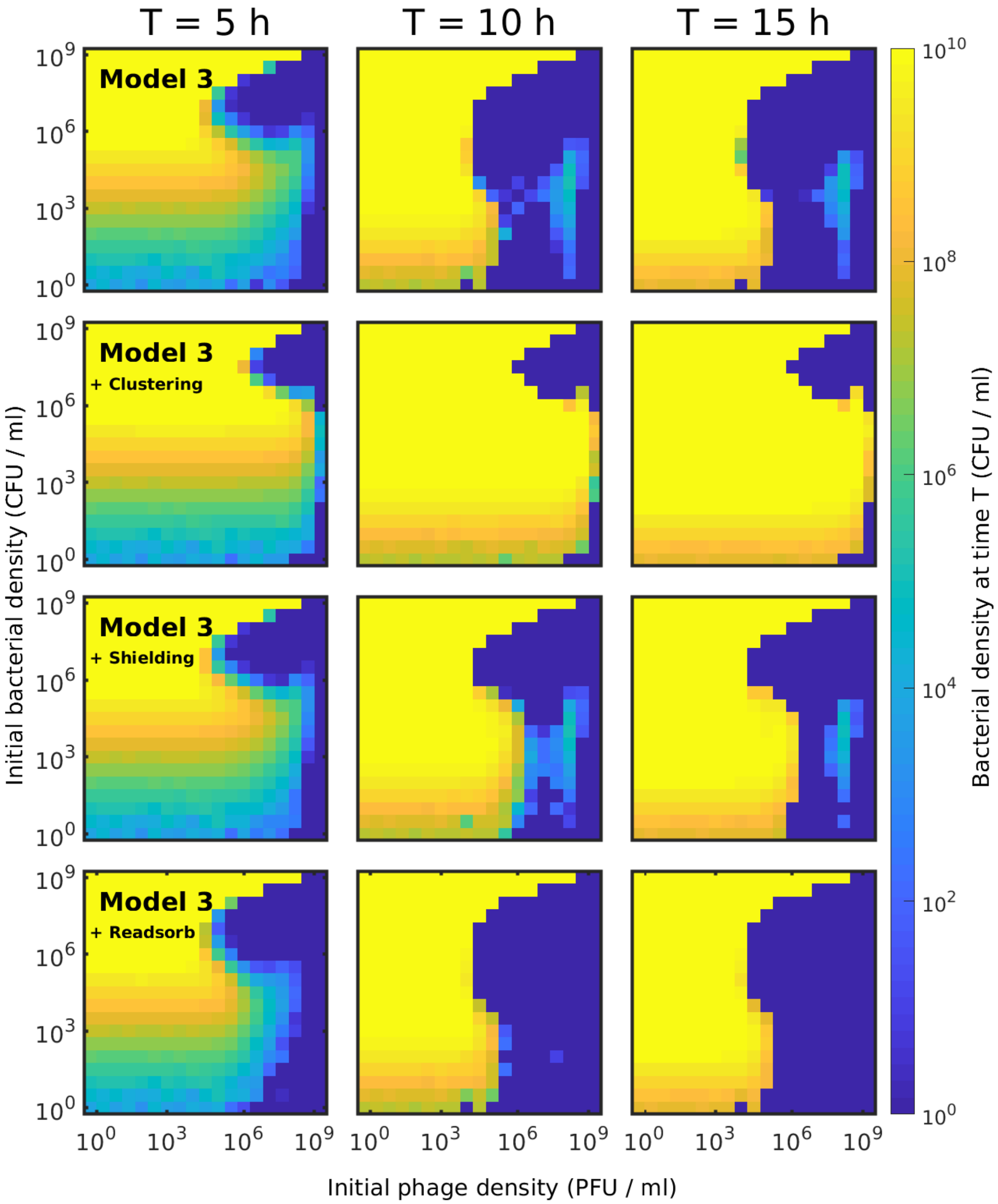

Figure S6. **Adding a single colony-level protection mechanism.** Our model contains several aspects of colony-level protection, and in this figure, we test their effect individually. Starting from model 3, the spatial model with time-delay (Fig. 4(c)), we add each mechanism of the colony-level protection separately. "Clustering" refers to the change in the adsorption term so it scales with bacterial density to the power $1/3$. "Shielding" refers to the reduced infection probability caused by phages having to move through already infected cells before reaching uninfected cells deeper in the colony. "Readsorb" refers a fraction of the released phages immediately adsorbing to the colony.

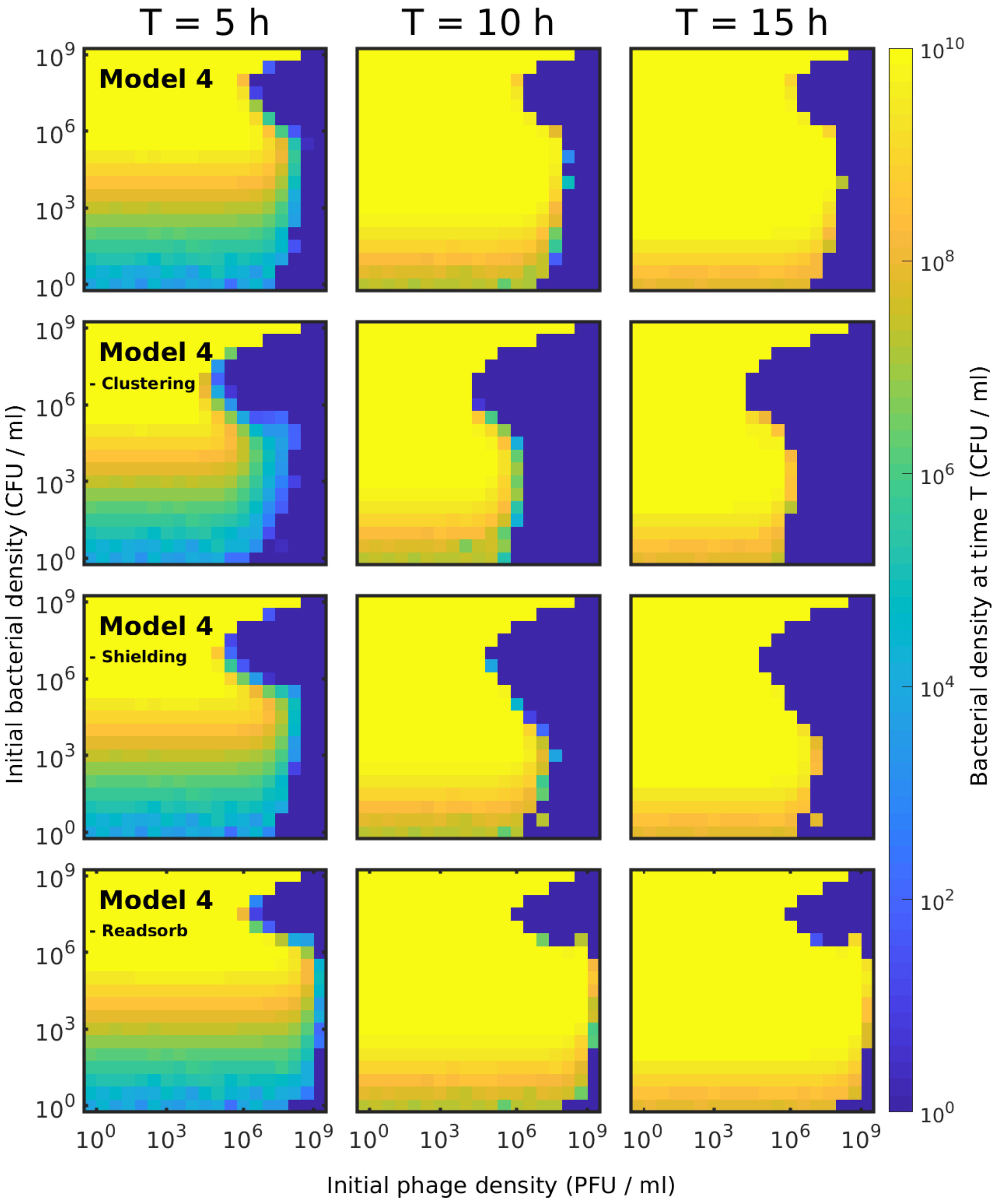

Figure S7. Removing a single colony-level protection mechanism.. Here we test how to colony-level protection mechanisms work pairwise. Using our model 4 (equation (4) of the main text) as a baseline we disable each mechanism one at a time, starting with the removal of the clustering effect. When clustering is disabled, we treat phage adsorption as in the well-mixed models where it is linear in the bacteria density. Next, we disable shielding effect and thus the phages can infect every cell with equal probability, and fewer phages are wasted by superinfecting bacteria. Finally, we remove the readsorption effect, meaning that released phages always escape the colony and become free phages.

6 The effect of spatial heterogeneity in the full model

The colony-level protection greatly increased the survival of the bacteria, and we now test whether these mechanisms have made the effects of including spatial variation redundant. We, therefore, run the model 4 (equation (4) of the main text) within a single box of size $\ell = 1$ cm and compare it to the result found in Fig. 4(d) (where $\ell = 200$ µm).

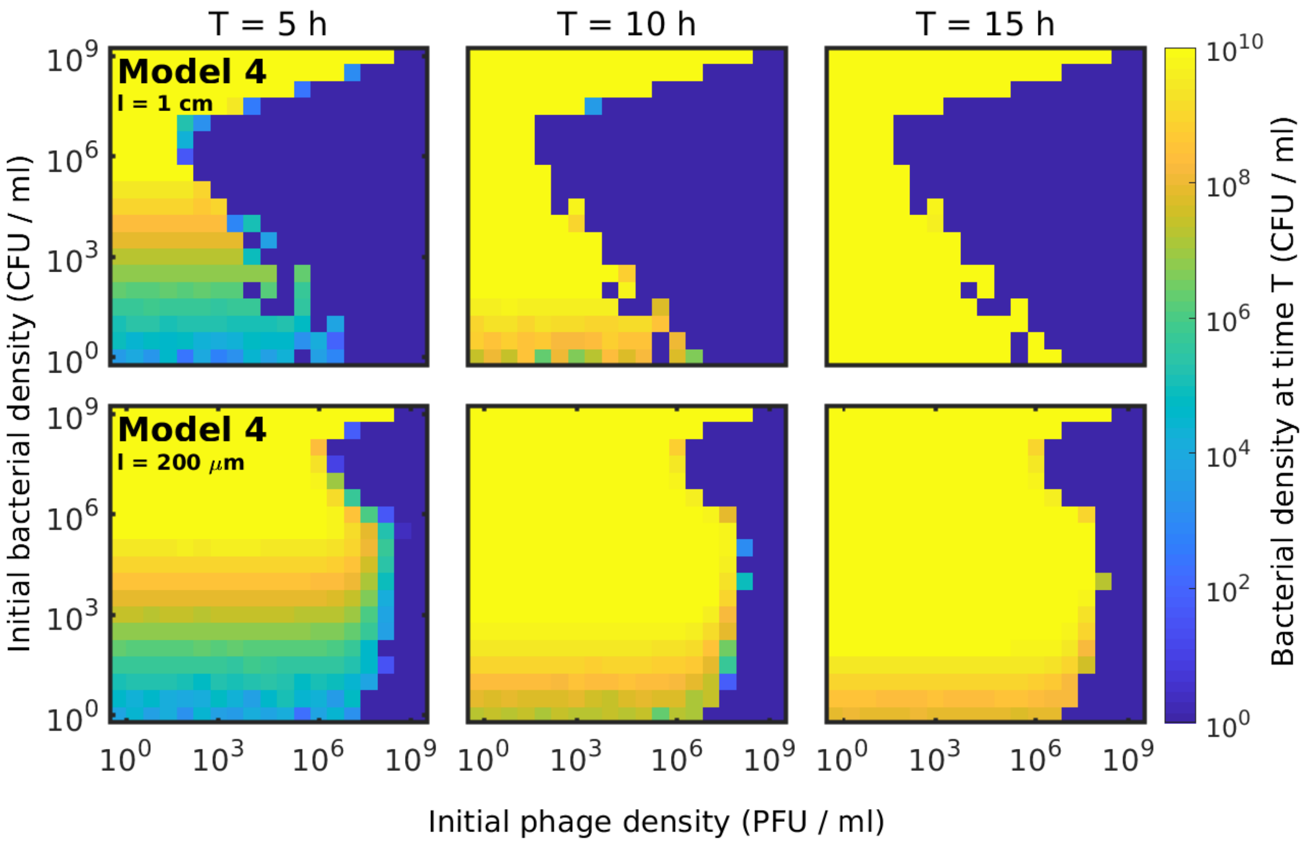

Figure S8. The effect of spatial heterogeneity. Our model 4 (equation (4) of the main text) contains several mechanism protecting the bacterial colonies. Here we test whether these mechanisms make the spatial variation in densities redundant. We therefore simulate our model without the addition of spatial variation ($\ell = 1$ cm), and with the inclusion of spatial variation ($\ell = 200$ µm).

From this test, we see that the moderating effects of spatial variation are still a large factor in allowing the bacteria to survive, even with the colony-level protection. One large factor of including spatial variation is to break the synchronization of events, which allows the bacterial colonies to have a distribution of sizes. Without variation, a single extremal event, such as an early adsorption event or a fast lysis event, will amplify quickly and can lead to a population collapse which would not happen otherwise. The heterogeneity introduced by space is therefore likely to be an important factor when modelling the interaction between bacteria and phage.

7 The distribution of the latency time

When introducing latency to model 2 (equation (2) of the main text) we use 10 internal states to delay the onset of lysis in the bacteria. The number of states to use is not completely arbitrary since the distribution of the latency time is strongly dependent on how many states are present.

To see why that is, we consider the n-step Poisson point process, where the rate of passing from any step S_i to the next S_{i+1} is equal to nq:

$$S_0 \xrightarrow{nq} S_1 \xrightarrow{nq} \ldots \xrightarrow{nq} S_n. \tag{S5}$$

If we start by considering the case of a single step ($n = 1$), the problem is simple. Here the rate of reaching the end state is just q and the probability that it takes time T to go from step S_0 to S_1 is simply $P_1(T) = q\exp(-qT)$.

Next, we can consider the case of two steps ($n = 2$), where the rate of going from one step to the next now is $2q$. The total time it takes to propagate to the end step is now $T = t_{01} + t_{12}$, where t_{01} and t_{12} is the time it takes to go from S_0 to S_1 and S_1 to S_2 respectively.

This leaves us with the inter-event distributions p_{01} and p_{12} which are distributions of the time it takes to go from step 0 to step 1, and from step 1 to step 2 respectively:

$$p_{01}(t_{01}) = 2q\exp(-2qt_{01}) \tag{S6}$$

$$p_{12}(t_{12}) = 2q\exp(-2qt_{12}) \tag{S7}$$

The probability of getting $T = t_{01} + t_{12}$ is then the product of the individual probabilities: $p_{01}(t_{01}) \cdot p_{12}(t_{12})$. We then obtain the distribution of T by integrating over all values of (t_{01}, t_{12}) that fulfill $T = t_{01} + t_{12}$:

$$P_2(T) = \int_0^T p(T - t_{12})p(t_{12})\mathrm{d}t_{12} \tag{S8}$$

Which we can explicitly solve:

$$P_2(T) = (2q)^2 \int_0^T \exp[-(2q)(T - t_{12})]\exp[-(2q)t_{12}]\,\mathrm{d}t_{12} \tag{S9}$$

$$= (2q)^2 \exp[-(2q)T] \int_0^T \mathrm{d}t_{12} \tag{S10}$$

$$= (2q)^2 T\exp[-(2q)T] \tag{S11}$$

Continuing this process gives the general result that the time to propagate the end step is Gamma distributed:

$$P_n(T) = \frac{1}{\Gamma(n)}(nq)^n T^{(n-1)} \exp[-(nq)T] \tag{S12}$$

This distribution has mean of $\langle T \rangle = \frac{1}{q}$ and a variance of $\sigma^2 = \frac{1}{nq^2}$. Note that by setting the rate to go from one step to the next to be nq the average time it takes to go through all steps becomes independent of n, while the shape of the distribution is strongly dependent on n. In our case, the average time $\langle T \rangle$ should be equal to the latency time τ meaning that $q = \frac{1}{\tau}$.

In Fig. S9 we show that using only a single internal state in model 2 (equation (2) of the main text) yields similar results to that of model 1 (equation (1) of the main text). The reason for this becomes clear when we consider the probability density functions for the latency time. When using a single internal state, the latency times will be exponentially distributed which means that the bulk of the lysis events happening soon after infections. In effect, this means that while the average latency time is now τ, the median latency is much shorter and the short latency events dominate the dynamics.

We see that going to 10 internal states gives a peaked distribution around τ meaning that most latency events will be properly delayed which in turn strongly effects the survival of the bacteria.

The choice of using 10 internal states rather than, e.g. 6 or 12, is that when using 10 states the coefficient of variation of the distribution becomes $\frac{1}{\sqrt{10}} \sim 30\%$.

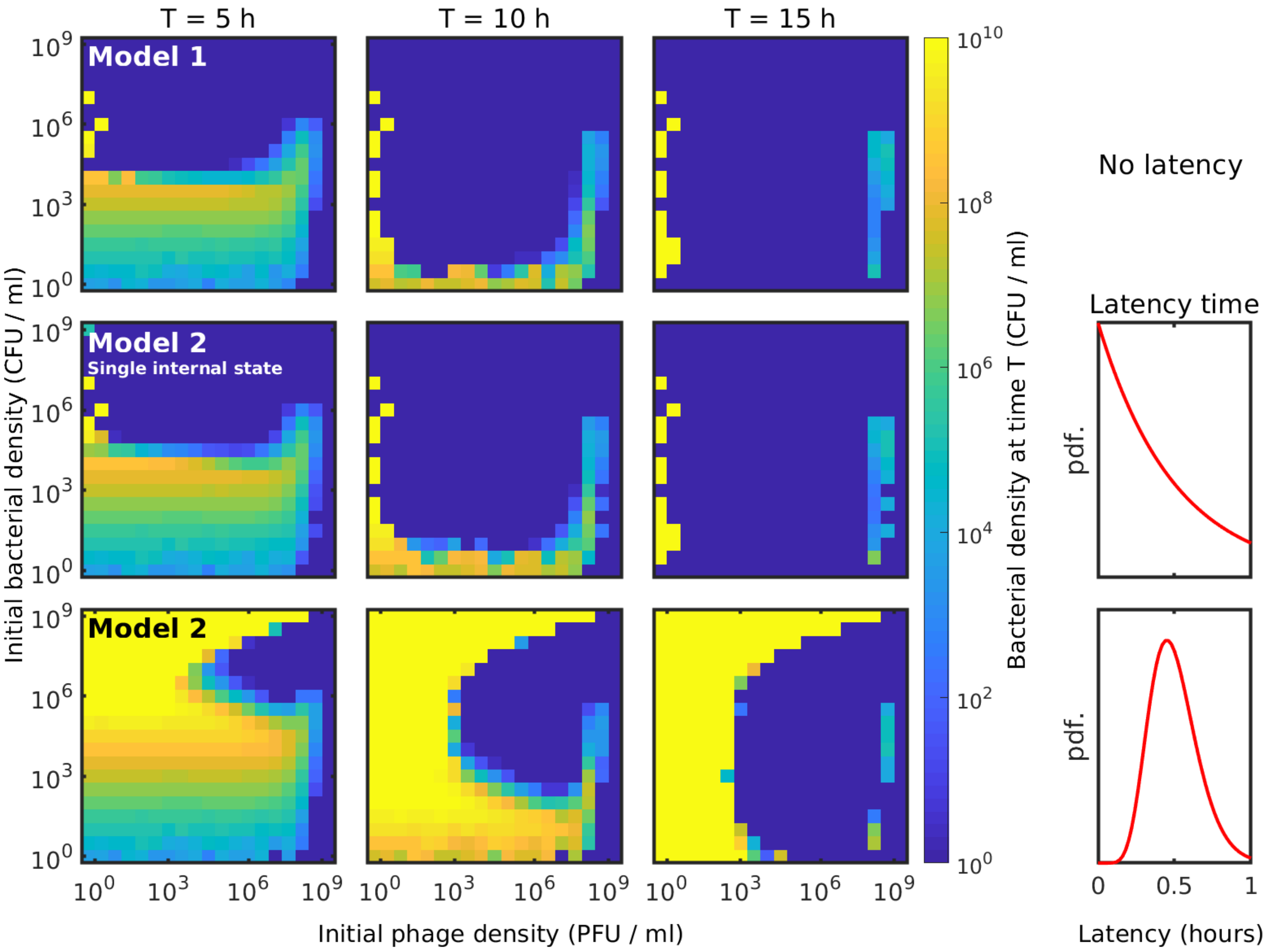

Figure S9. Latency time distributions. The latency between phage infection and cell lysis is an important feature of our investigation. Here we show how the shape of the latency time distribution (right-most panels) changes bacterial survival. We show bacterial survival in model 1, which is a well-mixed model without latency, and compare with model 2, a well-mixed model with latency. By changing the number of intermediate states in model 2, we change the distribution of latency times ranging from exponentially distributed (single internal state) to gamma distributed (10 internal sates).

8 The effect of latency in the full model

In this paper, we are interested mostly in how the spatial effects contribute to the survival of bacteria. However, we include a latency between phage infection and cell lysis in our model as we believe this to be an important aspect of phage virulence. In this section, we test how well spatial factors alone can protect the bacteria, and we, therefore, simulate models 3 and 4 in the limit where τ goes to 0 h. In Fig. S10, we compare the spatial models, with repeats of the same simulations without latency.

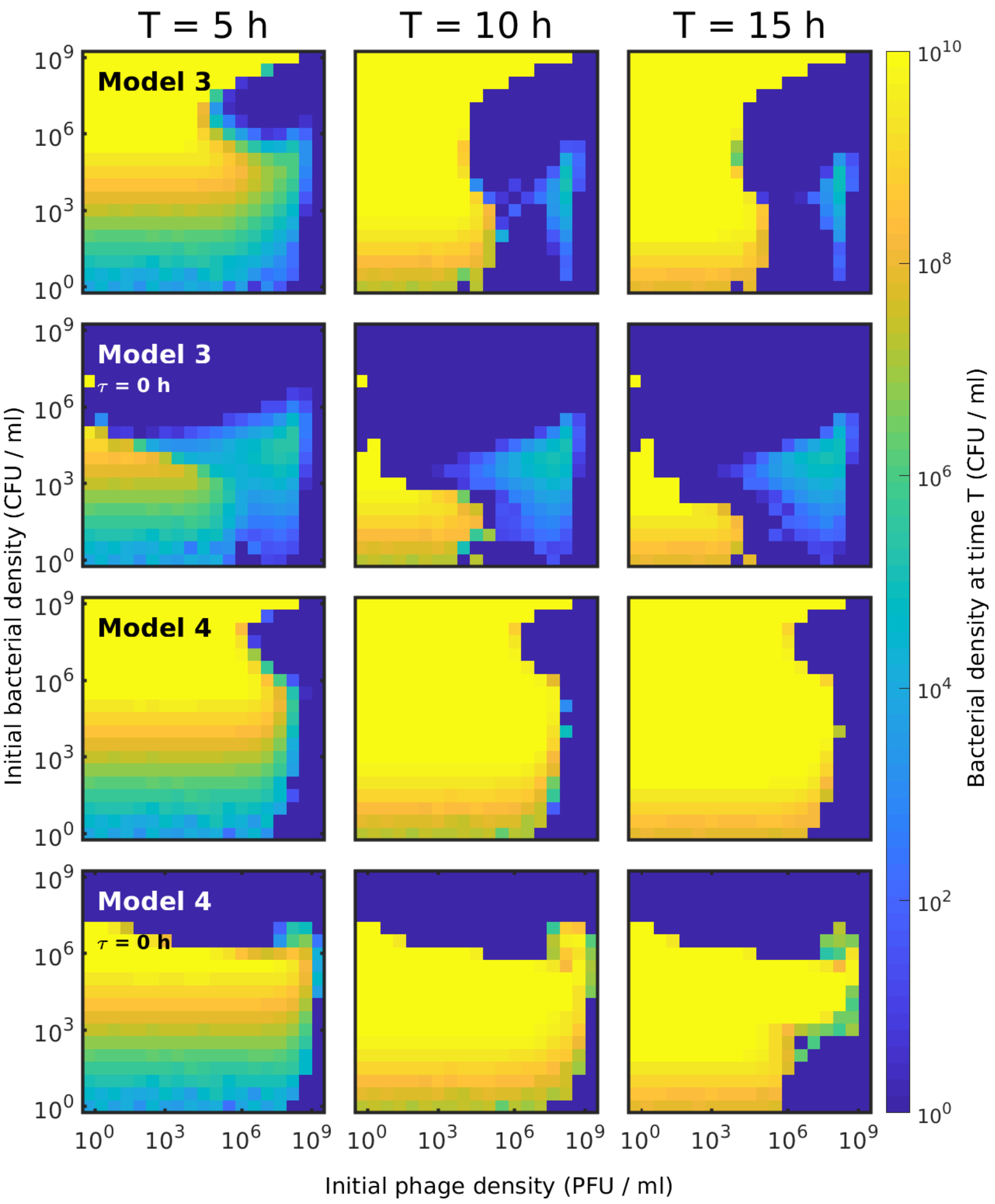

Figure S10. The effect of latency on bacterial survival. Here we highlight how the latency between infection in lysis moderates phage virulence in two of our models: (c) Spatial model with time-delay (equation (3) of the main text). (d) Spatial model with time-delay and colony-level protection mechanisms (equation (4) of the main text).

We see that spatial variation alone does provide refuge for the bacteria when the initial bacterial density is low. Here the clusters of bacteria are quite separated and the phages do not diffuse sufficiently fast to eliminate all bacteria before they consume the (bulk of the) nutrient. When including the formation of colonies, the bacteria survive at even large initial densities (of both phages and bacteria). However, the latency between infection and lysis is required to achieve survival at high initial densities since it allows the bacteria to consume the nutrient before the phage invasion takes hold.

9 Simulating experimental conditions

To validate model 4 (equation (4) of the main text), we use our simulations to mimic the experimental conditions of ref.[5]. Here the authors embed *Escherichia coli* in a layer of soft agar and allow the bacteria to form colonies before spraying the agar layer with virulent $P1_{vir}$ phages. The agar layer is thin (≈ 400 μm) and the initial bacteria are far away from each other (≈ 1 cm). This difference in length scales means that we increase the spatial resolution by setting $\ell = 0.1$ mm to better resolve the Z-axis. The increased resolution requires the nutrient to be solved on a time scale of $\Delta T = 5 \cdot 10^{-4}$ h ~ 2 s. The shorter time scale means that computation time increases significantly. To remedy this situation, we exploit the fact that phages move on average $\sim \sqrt{2 \cdot D_P \cdot 16\text{ h}} \approx 300$ μm during the experiment. We therefore only simulate the phages in an area of 10^3 μm $\times$ 10^3 μm $\times$ 400 μm centred on the colony.

9.1 Comparing ΔR with ζ

In the paper[5], the authors predict that a growing microcolony can survive a phage attack if its radius is above a certain threshold size R_c. By modelling the colony as a dense sphere of bacteria which consists of an inner core of uninfected cells surrounded by a shell of infected cells, they predict that the critical radius is: $R_c = 3[1 + (g\tau)^{-1}]\Delta R$. Where g is the growth rate of the bacteria, τ is the latency period for the phage, and ΔR is thickness of the shell of infected bacteria. From their measurement of $R_c \approx 25$ μm, we can estimate ΔR. Since the colony is small when survival is determined, we assume the nutrient depletion is negligible and set the effective bacterial growth rate $g = \lambda \frac{n_0}{n_0 + K} = \frac{60}{31}\frac{5}{6}$ h^{-1} $= 1.61$ h^{-1}. Rearranging their prediction yields the following expression:

$$\Delta R = \frac{R_c}{3(1 + (g\tau)^{-1})} \approx 5.5 \text{ μm}$$

Note that our model of the shielding function uses a similar logic. Here we assume that the colony is a densely packed sphere of bacteria, into which phages typically penetrate ζ layers before adsorbing to a target. Since *Escherichia coli* has a volume of $V_0 \sim 1$ μm^3, the parameter ζ gives the typical penetration depth: $\Delta R = \left(\frac{3V_0}{4\pi}\right)^{\frac{1}{3}} \zeta$. If we now equate the two expressions for ΔR, we get $\zeta \approx 8.3$.

9.2 Phage diffusion constant and the colony survival fraction

In our simulations of the experiment, we have observed that the survival of the bacterial colonies is strongly determined by the diffusion constant of the phages. This is due to the geometry of the experiment, bacteria were submerged in a layer of soft agar (~ 400 μm) and allowed to form colonies for some hours before the layer of soft agar was sprayed with phages. The rate of phage diffusion is in this scenario very important since it determines how long it takes for the bulk of the phage to diffuse deep enough into the agar to hit the colonies. The colonies experience a strong phage pressure early when the diffusion rate is fast, while the phage pressure is much lower early on when the diffusion rate is slow. This is important since the colony-level protection mechanisms are size-dependent and the difference in timing allows the colonies to grow to a much larger size before the phage invasion arrives. Consequently, the value of the phage diffusion constant has an impact on which value of ζ is needed to reproduce the experimental results.

In Fig. S11, we show the fraction of surviving colonies for two phage diffusion constants ($D_P = 10000$ μm^2/h ~ 3 μm^2/s and $D_P = 3000$ μm^2/h ~ 1 μm^2/s).

We see that when the diffusion constant for the phage is large, the shielding parameter ζ has to be smaller to compensate for the increased phage density in the vicinity of the colony.

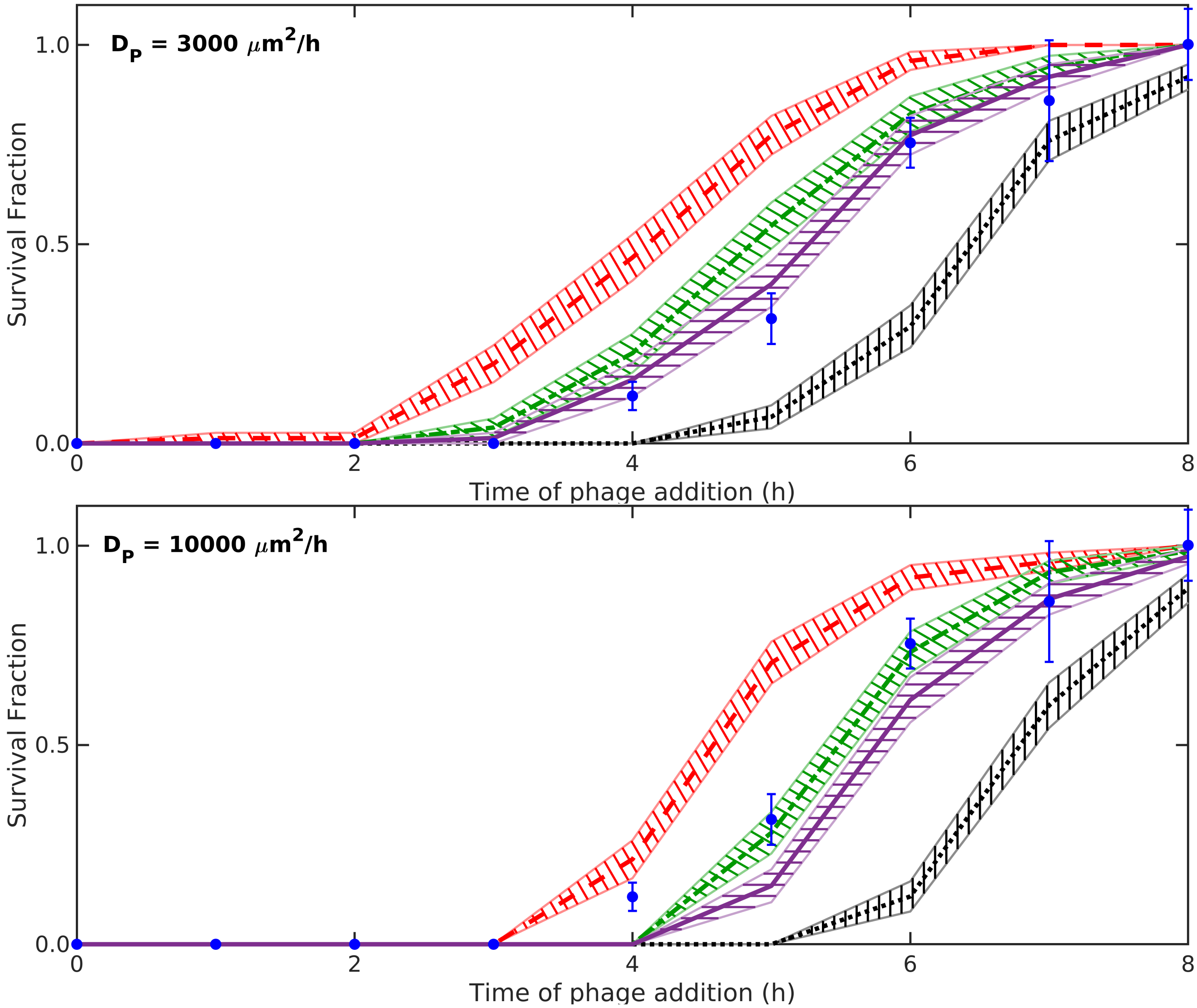

Figure S11. **Phage diffusion constant and colony survival.** The fraction of colonies which grows to visible size in experiments (blue error-bars) and the fraction of simulated colonies ($n = 75$) which grow to "visible" size for $\zeta = 2.5$ (red dashed line), $\zeta = 5$ (green dash-dotted line), $\zeta = 10$ (purple fully drawn line), and when colony-level protections are disabled (black dotted line). The hatched area corresponds to the standard error on the mean. See the main text for more details.

10 Implementation details

In this section, we present the default parameters used throughout the paper (see Table S1), as well as a breakdown of the how the equations have been interpreted as describing the rates at which events occur.

Parameter	Value	Unit	Comment	Reference/Additional comment
ΔT	$2 \cdot 10^{-3}$	h	Time increment size	See Section 11 for derivation
L	10^4	μm	Length of simulated space	Corresponding to a 1 mL volume
ℓ	200	μm	Length of each lattice point	Lattice is $50 \times 50 \times 50$
λ	2	1/h	Maximal bacterial growth rate	Cell doubles every half hour
n_0	10^9	1/mL	Available nutrient (at $t = 0$ h)	
K	n_0 / 5	1/mL	Michaels-Menten constant	Half growth rate at 20% nutrient level
α	0.5		Readsorption probability	
β	100		Burst size	
δ	0.1	1/h	Phage decay rate	Value in ocean[6]
η	$10^4/\ell^3$	1/h	Adsorption rate	10^{-8} mL/h<br>Value in liquid culture[7]
τ	0.5	h	Lysis latency time	Value in liquid culture[7]
ζ	1		Surface permeability	# of bacterial layers the phage can pass through
D_P	10^4	μm^2/h	Phage diffusion constant	~ 3 μm^2/s<br>T7 diffusion[3]: $1.17 \cdot 10^{-2}$ mm^2/h ~ 3 μm^2/s<br>λ diffusion[2]: 6.2×10^{-8} cm^2/s ~ 6 μm^2/s
D_B	0	μm^2/h	Bacterial diffusion constant	Immotile on the length-scale we consider
D_n	$2.5 \cdot 10^6$	μm^2/h	Nutrient diffusion constant	$\sim 0.75 \cdot 10^9$ m^2/s<br>Value in liquid $\sim 0.5 \cdot 10^{-9}$ m^2/s – 10^{-9} m^2/s[8].

Table S1. The default values used in the simulations.

We first consider the equation which describes the dynamics of the nutrient in the system:

$$\frac{\partial n}{\partial t} = -\lambda B \frac{n}{n+K} + D_n \nabla^2 n$$

This contains two terms, a term which comes from the growth of bacteria $\lambda B \frac{n}{n+K}$ and a term which gives diffusion of nutrient $D_n \nabla^2 n$. As mentioned in the main text, the diffusion of nutrient is treated deterministically, and therefore can readily be implemented by a discrete version of the Laplace operator (forward time, central space) using a 7-point stencil. We interpret the term which describes the growth of bacteria as the rate at which new bacteria are formed. We therefore have an event (single bacterium dividing) and a rate ρ_1 of the event ($\rho_1 = \lambda \frac{n}{n+K}$). From the rate, we compute the probability p_1 that a bacterium divides in the given time step as $p_1 = \rho_1 \cdot \Delta T$. We then draw the number of events N_1 which occur from a Poisson distribution with mean $p_1 \cdot B$. For each birth event that occurs two things happen: 1) one unit of nutrient is being consumed (corresponding to the minus sign in the above equation) and 2) one new bacteria is introduced.

If we now consider the equation which gives the dynamics of the uninfected bacteria:

$$\frac{\partial B}{\partial t} = \lambda B \frac{n}{n+K} - S(B, I, n_c, \zeta) \left[\eta \left(\frac{B+I}{n_c} \right)^{1/3} n_c P + \alpha \beta \frac{10}{\tau} I_{10} \frac{n}{n+K} \right]$$

We see the implementation of the birth event describes the first term in the equation.

The remaining term is: $S(B, I, n_c, \zeta) \left[\eta \left(\frac{B+I}{n_c} \right)^{1/3} n_c P + \alpha \beta \frac{10}{\tau} I_{10} \frac{n}{n+K} \right]$. This terms describes the number of uninfected bacteria which are being hit by phages. The phages come from two sources: free phages P and from bacteria which undergo

lysis. The term can be interpreted as a series of events, each with different rates. First, we consider the event: free phages hitting the colonies. A free phage will hit a colony at a rate of $\rho_2 = \eta\left(\frac{B+I}{n_c}\right)^{1/3}$. Since we have n_c colonies, the phage will hit any colony with probability $p_2 = 1-(1-\rho_2\cdot\Delta T)^{n_c}$. We can get the number of phages N_2 which hits colonies by drawing a number from the Poisson distribution with mean $p_2\cdot P$. Similarly, we can compute the number of bacteria which undergo lysis by considering the rate of which bacteria lyse: $\rho_3 = \frac{10}{\tau}\frac{n}{n+K}$. And compute the probability of lysis during the time step: $p_3 = \rho_3\cdot\Delta T$, and then draw the number of lysing bacteria as N_3 from a Poisson distribution with mean $p_3\cdot I_{10}$. For each lysis event that occurs β new phages are released, of which a fraction α readsorbs to the colonies. We can then compute the number of re-adsorbed phages which hit the colony: $N_4 = \alpha\cdot\beta\cdot N_3$.

For each of these events (N_2+N_4), a phage has adsorbed to a bacterium and deployed its genetic material and we remove them from the simulation. Next, we compute how many of these phages has slipped through the surface to reach the uninfected bacteria. Here we need a careful implementation to ensure that the algorithm is insensitive to the time step size ΔT. The first phage that hits the colony has a probability $p_5^1 = S(B,I,n_c,\zeta)$ of hitting the uninfected bacteria within. If this phage successfully hits the uninfected bacteria within, we increase the number N_5 by one. This number starts at zero and counts the number of adsorption events that result in new infected states. The second phage will have probability $p_5^2 = S(B-N_5, I+N_5, n_c,\zeta)$ of hitting the uninfected bacteria within the colony, since now the number of uninfected and infected bacteria has changed. Using this iterative method, we update he value of N_5 separately for each adsorption event.

Next, we consider the equations of the infected bacteria:

$$\begin{aligned}\frac{\partial I_1}{\partial t} &= S(B,I,n_c,\zeta)\left[\eta\left(\frac{B+I}{n_c}\right)^{1/3} n_c P + \alpha\beta\frac{10}{\tau}I_{10}\frac{n}{n+K}\right] - \frac{10}{\tau}I_1\frac{n}{n+K}\\ \frac{\partial I_k}{\partial t} &= \frac{10}{\tau}\frac{n}{n+K}\left(I_{k-1}-I_k\right) \quad i = 2,3,...,10\end{aligned}$$

At this point, we have already computed the number of uninfected bacteria hit by phages (N_5), and we have therefore covered the influx term in the equation of for I_1. The remaining terms all describe events where an infected bacterium moves from one stage to the next stage. All of these events have the same rate as we have seen from I_{10}, namely the rate ρ_3. We can then compute the number of bacteria which moves out from state I_k as N_{k+6} drawn from a Poisson distribution with mean $p_3\cdot I_k$.

Finally, we can consider the equation that describes the phages:

$$\frac{\partial P}{\partial t} = (1-\alpha)\beta\frac{10}{\tau}I_{10}\frac{n}{n+K} - \delta P - \eta\left(\frac{B+I}{n_c}\right)^{1/3} n_c P + D_P\nabla^2 P$$

Here we have computed the number of bacteria lysing N_3, and the first term in the equation has therefore already been covered. The second term $-\delta P$, describes the number of phages which decay over time. Here we can compute the number of phages which decay, N_{16}, from the Poisson distribution with mean $\delta\cdot\Delta T\cdot P$. The third term has been covered by N_2 when we computed the number phages which hit the colonies. Finally, we have the diffusion term $D_P\nabla^2 P$, which we treat as random walk where each phage has a probability $2\frac{D_P\Delta T}{\ell^2}$ of jumping to a neighbour point. By drawing from a Poisson distribution, we can compute the flux of phages into and out of the grid point.

11 Von Neumann stability analysis of the 3D FTCS scheme

In three dimensions the diffusion equation takes the form:

$$\frac{\partial u}{\partial t} = D\left(\frac{\partial^2 u}{\partial x^2} + \frac{\partial^2 u}{\partial y^2} + \frac{\partial^2 u}{\partial z^2}\right) \tag{S13}$$

Since we are solving the equation on a discrete 3D lattice we discretize u as:

$$u^t_{l,m,n} = u(x_0 + l\Delta x,\ y_0 + m\Delta y,\ z_0 + n\Delta z, t_0 + t\Delta T)$$

The dimensions of our lattice is symmetric which means $\Delta x = \Delta y = \Delta z = \Delta$. We use the "Forward Time Central Space" (FTCS) stencil to approximate equation (S13) which yields the following discrete form:

$$\frac{u^{t+\Delta T}_{l,m,n} - u^t_{l,m,n}}{\Delta T} = D\frac{u^t_{l+1,m,n} + u^t_{l-1,m,n} + u^t_{l,m+1,n} + u^t_{l,m-1,n} + u^t_{l,m,n+1} + u^t_{l,m,n-1} - 6u^t_{l,m,n}}{\Delta^2} \tag{S14}$$

Now we insert the Fourier mode $v_k = c_k \exp(i\vec{k}\cdot\vec{r})$ into the discrete approximation (S14) and obtain:

$$\frac{v^{t+1}_k - v^t_k}{\Delta T} = \frac{Dv^t_k}{\Delta^2}\left(\exp(ik_x\Delta) + \exp(-ik_x\Delta) + \exp(ik_y\Delta) + \exp(-ik_y\Delta) + \exp(ik_z\Delta) + \exp(-ik_z\Delta) - 6\right)$$

Which we simplify to:

$$\frac{v^{t+1}_k - v^t_k}{\Delta T} = \frac{2Dv^t_k}{\Delta^2}\left(\cos(k_x\Delta) + \cos(k_y\Delta) + \cos(k_z\Delta) - 3\right)$$

By isolating v^{t+1}_k we get:

$$v^{t+1}_k = \left[1 - \frac{2D\Delta T}{\Delta^2}\left(3 - \cos(k_x\Delta) - \cos(k_y\Delta) - \cos(k_z\Delta)\right)\right] v^t_k$$

Stability is then obtained when the amplitude of the mode does not increase over time, i.e. when:

$$1 - \frac{2D\Delta T}{\Delta^2}\left(3 - \cos(k_x\Delta) - \cos(k_y\Delta) - \cos(k_z\Delta)\right) \leq 1$$

Since cos is bounded by $[-1, 1]$, we can simplify our requirement further:

$$\left|1 - 12\frac{D\Delta T}{\Delta^2}\right| \leq 1$$

Which means that if $12\frac{D\Delta T}{\Delta^2} \leq 2$ stability is obtained. That is, the FTCS scheme is stable if we impose:

$$\frac{D\Delta T}{\Delta^2} \leq \frac{1}{6} \tag{S15}$$

References

1. Gudowska-Nowak, E., Lindenberg, K. & Metzler, R. Preface: Marian Smoluchowski's 1916 paper—a century of inspiration. *J. Phys. A: Math. Theor.* **50**, 380301, DOI: 10.1088/1751-8121/aa8529 (2017).
2. Moldovan, R. & Wu, X. L. On Kinetics of Phage Adsorption. *Biophys. J.* **93**, 303–315, DOI: 10.1529/biophysj.106.102962 (2007).
3. Payne, P., Geyrhofer, L., Barton, N. H. & Bollback, J. P. CRISPR-based herd immunity can limit phage epidemics in bacterial populations. *eLife* **7**, 1–33, DOI: 10.7554/elife.32035 (2018).
4. Tavaddod, S., Charsooghi, M. A., Abdi, F., Khalesifard, H. R. & Golestanian, R. Probing passive diffusion of flagellated and deflagellated Escherichia coli. *The Eur. Phys. J. E* **34**, 16, DOI: 10.1140/epje/i2011-11016-9 (2011).
5. Eriksen, R. S., Svenningsen, S. L. L., Sneppen, K. & Mitarai, N. A growing microcolony can survive and support persistent propagation of virulent phages. *Proc. Natl. Acad. Sci.* **115**, 337–342, DOI: 10.1073/pnas.1708954115 (2018). 1703.08755.
6. Noble, R. T. & Fuhrman, J. A. Virus decay and its causes in coastal waters. *Appl. environmental microbiology* **63**, 77–83 (1997).
7. De Paepe, M. & Taddei, F. Viruses' life history: Towards a mechanistic basis of a trade-off between survival and reproduction among phages. *PLoS Biol.* **4**, 1248–1256, DOI: 10.1371/journal.pbio.0040193 (2006).
8. Ribeiro, A. C. *et al.* Binary mutual diffusion coefficients of aqueous solutions of sucrose, lactose, glucose, and fructose in the temperature range from (298.15 to 328.15) K. *J. Chem. Eng. Data* **51**, 1836–1840, DOI: 10.1021/je0602061 (2006).
